# Ultrafast and long-time excited state kinetics of an NIR-emissive vanadium(iii) complex I: synthesis, spectroscopy and static quantum chemistry†‡

**DOI:** 10.1039/d1sc02137k

**Published:** 2021-07-15

**Authors:** Matthias Dorn, Jens Kalmbach, Pit Boden, Ayla Kruse, Chahinez Dab, Christian Reber, Gereon Niedner-Schatteburg, Stefan Lochbrunner, Markus Gerhards, Michael Seitz, Katja Heinze

**Affiliations:** Department of Chemistry, Johannes Gutenberg University of Mainz Duesbergweg 10-14 55128 Mainz Germany katja.heinze@uni-mainz.de; Institute of Inorganic Chemistry, University of Tübingen Auf der Morgenstelle 18 72076 Tübingen Germany; Department of Chemistry and Research Center Optimas, Technical University Kaiserslautern Erwin-Schrödinger-Straße 67663 Kaiserslautern Germany; Institute for Physics, Department of Life, Light and Matter, University of Rostock 18051 Rostock Germany; Département de chimie, Université de Montréal Montréal Québec H3C 3J7 Canada

## Abstract

In spite of intense, recent research efforts, luminescent transition metal complexes with Earth-abundant metals are still very rare owing to the small ligand field splitting of 3d transition metal complexes and the resulting non-emissive low-energy metal-centered states. Low-energy excited states decay efficiently non-radiatively, so that near-infrared emissive transition metal complexes with 3d transition metals are even more challenging. We report that the heteroleptic pseudo-octahedral d^2^-vanadium(iii) complex VCl_3_(ddpd) (ddpd = *N*,*N*′-dimethyl-*N*,*N*′-dipyridine-2-yl-pyridine-2,6-diamine) shows near-infrared singlet → triplet spin–flip phosphorescence maxima at 1102, 1219 and 1256 nm with a lifetime of 0.5 μs at room temperature. Band splitting, ligand deuteration, excitation energy and temperature effects on the excited state dynamics will be discussed on slow and fast timescales using Raman, static and time-resolved photoluminescence, step-scan FTIR and fs-UV pump-vis probe spectroscopy as well as photolysis experiments in combination with static quantum chemical calculations. These results inform future design strategies for molecular materials of Earth-abundant metal ions exhibiting spin–flip luminescence and photoinduced metal–ligand bond homolysis.

## Abstract 2

Vanadium is an abundant and cheap metal but near-infrared luminescent vanadium complexes are extremely rare with largely unexplored photophysics and photochemistry. We delineate the photodynamics of VCl_3_(ddpd) to infer novel design strategies.

## Graphical abstract

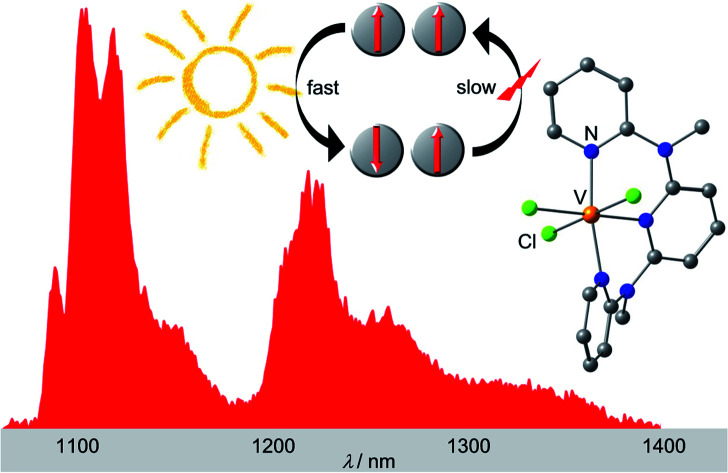

## Introduction

The control of photophysical properties of transition metal complexes by chemical means, especially for applications in lighting, imaging, sensing, photonics, dye sensitised solar cells, phototherapy or photocatalysis, is a very active research field.^1–8^ However, most applications rely on noble metal complexes with d^6^ or d^8^ electron configurations such as ruthenium(ii), iridium(iii) or platinum(ii).^9^ The success of these precious metals in photophysics and photochemistry can be ascribed, among other beneficial features, to their intrinsically large ligand field splitting^10^ and their large spin–orbit coupling (SOC) constants *ζ* ≫ 1000 cm^−1^.^11^ Finally, these properties enable the efficient population of the photoactive and luminescent long-lived triplet metal-to-ligand charge transfer (^3^MLCT) states as lowest excited states after intersystem crossing (ISC).^12^

Aiming at a sustainable future photochemistry less dependent on rare and precious metals, Earth-abundant metals are currently heavily explored and novel concepts have been put forward^13–18^ including some second row metals,^19–22^ but in particular the first row transition metals.^13–18^ The 3d transition metals possess a weaker ligand field splitting^10^ and smaller SOCs^11^ posing severe challenges to the design of the excited state landscape,^14^ yet several recent breakthroughs have been reported, *e.g.* on copper(i),^23,24^ nickel(0,ii),^25,26^ cobalt(iii),^27^ iron(ii,iii),^28–31^ chromium(0/iii)^32–35^ and vanadium(iii).^36^ Beyond the conventionally exploited MLCT excited states,^12^ LMCT states of the low-spin d^5^ electron configuration of iron(iii)^18^ and spin–flip states of the d^3^ electron configuration of chromium(iii)^15^ are emerging as novel paradigmatic excited states useful for photoapplications.

The currently most successful spin–flip emitters are based on the so-called molecular ruby motif, *e.g.* in [Cr(ddpd)_2_]^3+^, with tridentate pyridine-type ligands forming six-membered chelate rings (ddpd = *N*,*N*′-dimethyl-*N*,*N*′-dipyridine-2-yl-pyridine-2,6-diamine).^33,34,37^ Applications already emerged in the areas of sensing,^38–40^ photocatalysis,^37^ photodynamic therapy,^41^ photon upconversion^42^ and the generation of circularly polarised emission.^43–45^

**Chart 1:**
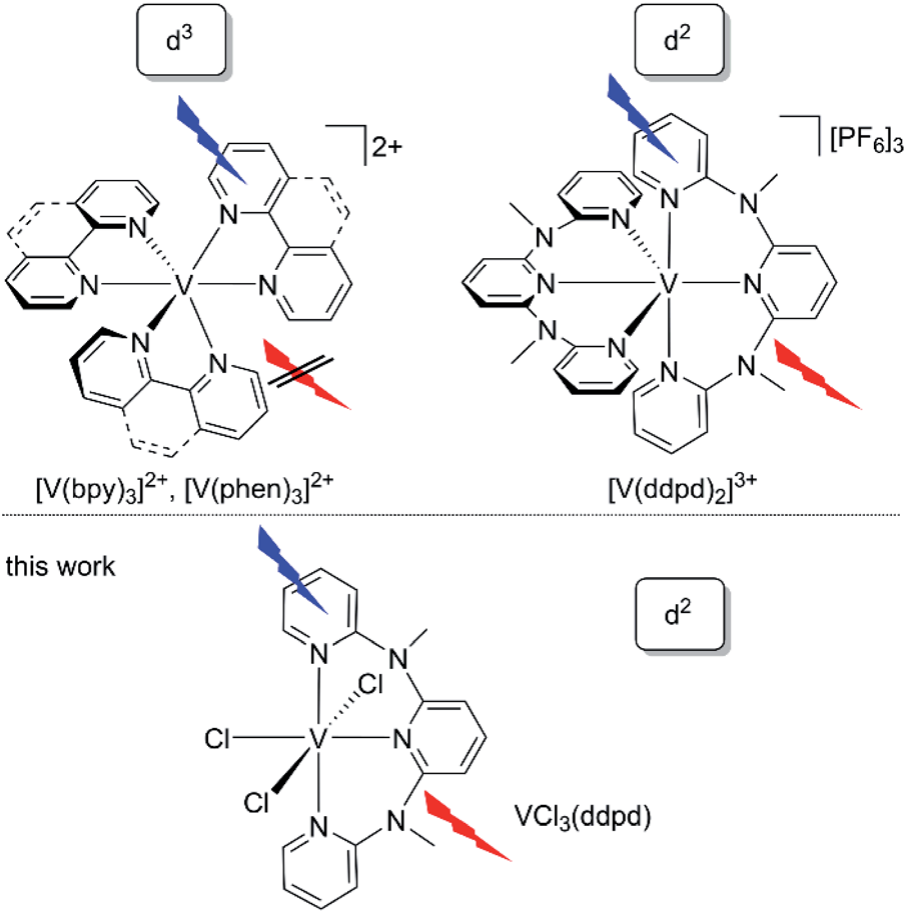
Structures of vanadium(ii) and luminescent vanadium(iii) polypyridine complexes.

Very recently, vanadium started to spark interest as potential novel near-infrared (NIR) luminophore^46^ motivated by its high natural abundance and complementary properties to the chromium(iii) spin–flip luminophores and sensitisers.^36,47^ Rappé and Damrauer demonstrated, that the d^3^-vanadium(ii) electron configuration in the well-known [V(bpy)_3_]^2+^ and [V(phen)_3_]^2+^ complexes^48^ (Chart 1, bpy = 2,2′-bipyridine, phen = 1,10-phenanthroline) leads to the population of non-luminescent excited doublet states with mixed ^2^MC/^2^MLCT character within 2.5–3 ps after excitation.^47^ The lifetimes of the mixed excited states of [V(bpy)_3_]^2+^ and [V(phen)_3_]^2+^ are 0.43 ns and 1.6 ns, respectively.^47,49^ Yet, emission from these vanadium(ii) complexes has not been observed at wavelengths shorter than 1600 nm even at low temperature.^47^

Analogous to the intra- and interconfigurational electronic transitions derived from the d^3^ electron configuration (^4^T_2_ and ^2^E/^2^T_1_), the d^2^ electron configuration with a ^3^T_1_ ground state in octahedral symmetry provides low-energy spin–flip singlet states (^1^E/^1^T_2_) and triplet states (^3^T_2_) with respective intra- and interconfigurational transitions.^50^ This electron configuration is realised, for example, in octahedral vanadium(iii) complexes and solid state materials with vanadium(iii) in octahedral sites.^51–53^ The electronic structure of basic d^2^-vanadium(iii) complexes, *e.g.* [V(H_2_O)_6_]^3+^ and [V(urea)_6_]^3+^, including the ground state splitting of the orbitally degenerate ^3^T_1_ ground state and the zero-field splitting, has been obtained from Raman, luminescence, and high-frequency high-field electron paramagnetic resonance spectroscopies.^51–53^ A particularly interesting application of d^2^-luminescent materials is upconversion based on sequential ground state and excited state absorption,^54^ as has been shown for example with d^2^-titanium(ii) solid state materials such as MgCl_2_:Ti.^54^ Upconversion with molecular complexes has been demonstrated utilizing d^3^-chromium(iii) complexes.^42,55^ The five-coordinate d^2^-vanadium(iii) complex V((C_6_F_5_)_3_tren)(CN^*t*^Bu) emits at 1240 nm in the solid state and frozen solution and was suggested as optically addressable molecular quantum bit candidate ((C_6_F_5_)_3_tren = 2,2′,2′′-tris[(pentafluorphenyl)amino] triethylamine).^56^ Consequently, the advancement of emissive molecular materials exploiting the d^2^ electronic configuration would be very valuable for diverse photonic applications such as NIR emission, upconversion and quantum technology.

The d^2^-vanadium(iii) polypyridine complex [V(ddpd)_2_]^3+^ emits in the NIR (1100 nm), observed for the first time even at room temperature in solution (Chart 1).^36^ Its phosphorescent singlet state with a lifetime of 0.79 μs/8.8 μs (93%/7%; 77 K in butyronitrile glass) is populated within picoseconds after excitation. Yet, the efficiency of the population transfer to the singlet states by ISC is rather small as confirmed by non-adiabatic molecular dynamics calculations.^36^ Unexpectedly, and in contrast to the analogous chromium(iii) spin–flip emitters, the decay of the phosphorescent spin–flip states of [V(ddpd)_2_]^3+^ is insensitive to ligand deuteration, in spite of the significant spectral overlap of the NIR emission with the second aromatic C–H overtone ν^3^_CH_ of the ligand. This suggests that other non-radiative decay pathways are more relevant than the multiphonon relaxation involving high-energy C–H oscillators^57^ in this particular case.^36^

A profound understanding of the decisive excited states and the excited state dynamics of these polypyridine vanadium(iii) chromophores on ultrafast (population of emissive states) and slow timescales (depopulation of emissive states) is lacking. To better understand the novel class of d^2^-spin–flip luminophores based on vanadium(iii) with respect to the population and decay of the emissive spin–flip states, we selected the chlorido vanadium(iii) complex VCl_3_(ddpd) (Chart 1)^58^ for a detailed study of the photodynamics at ultrashort (sub-picosecond) to microsecond timescales (Chart 1).

Spin–orbit effects are weak in vanadium(iii) complexes based on the lower intensity of the singlet transitions compared to the triplet bands by more than three orders of magnitude.^59^ Our choice of molecular system is guided by two considerations: (i) the slow ISC rate defined by the small SOC constant of vanadium(iii) (*ζ* ≈ 210/206/220 cm^−1^)^11,60,61^ can increase due to the influence of the coordinated chlorido ligands with their higher SOC constant (*ζ* ≈ 547 cm^−1^)^61^ as compared to nitrogen (*ζ* ≈ 76 cm^−1^).^61^ (ii) The symmetry reduction by using different ligand types can relax Laporte's rule^62^ and increase the radiative rate from metal-centred spin–flip states.^35^

We report here that the heteroleptic VCl_3_(ddpd)^58^ complex is NIR-emissive at room temperature (Chart 1). We undertook a detailed photophysical study using Raman spectroscopy, variable temperature and variable pressure steady-state photoluminescence spectroscopy, fs-transient absorption spectroscopy, time-resolved photoluminescence and variable temperature step-scan FTIR spectroscopy to cover the ground state splitting, as well as the ultrafast and slow time regimes of the excited state kinetics. To elucidate whether non-radiative relaxation of the luminescent singlet states *via* aromatic C–H overtones (C–H_ar_) plays a significant role for the non-radiative relaxation in this particular case, the deuterated complex VCl_3_(ddpd-[D_17_]) was prepared and studied for comparison. The electronic structures of ground and excited states at the ground state geometry were described utilizing relativistic two-component time-dependent density functional theory (TDDFT) and CASSCF-NEVPT2 calculations (NEVPT2 = N-electron valence state perturbation theory to second-order). A detailed kinetic model of the excited state dynamics will be derived by trajectory surface hopping simulations within a linear vibronic coupling model in the accompanying paper.^63^

## Results and discussion

The vanadium(iii) complex VCl_3_(ddpd-[D_0_]) has been prepared as reported from VCl_3_(solv)_3_ (solv = CH_3_CN or THF)^64^ and the ligand ddpd-[D_0_]^65^ as poorly soluble orange coloured complex.^58^ Its structure and magnetic properties in the solid state have been reported.^58^ Yet, luminescence, ultrafast and slow excited-state dynamics as well as temperature and pressure effects on the decisive ground and excited states and the excited state kinetics remained unexplored.

**Fig. 1:**
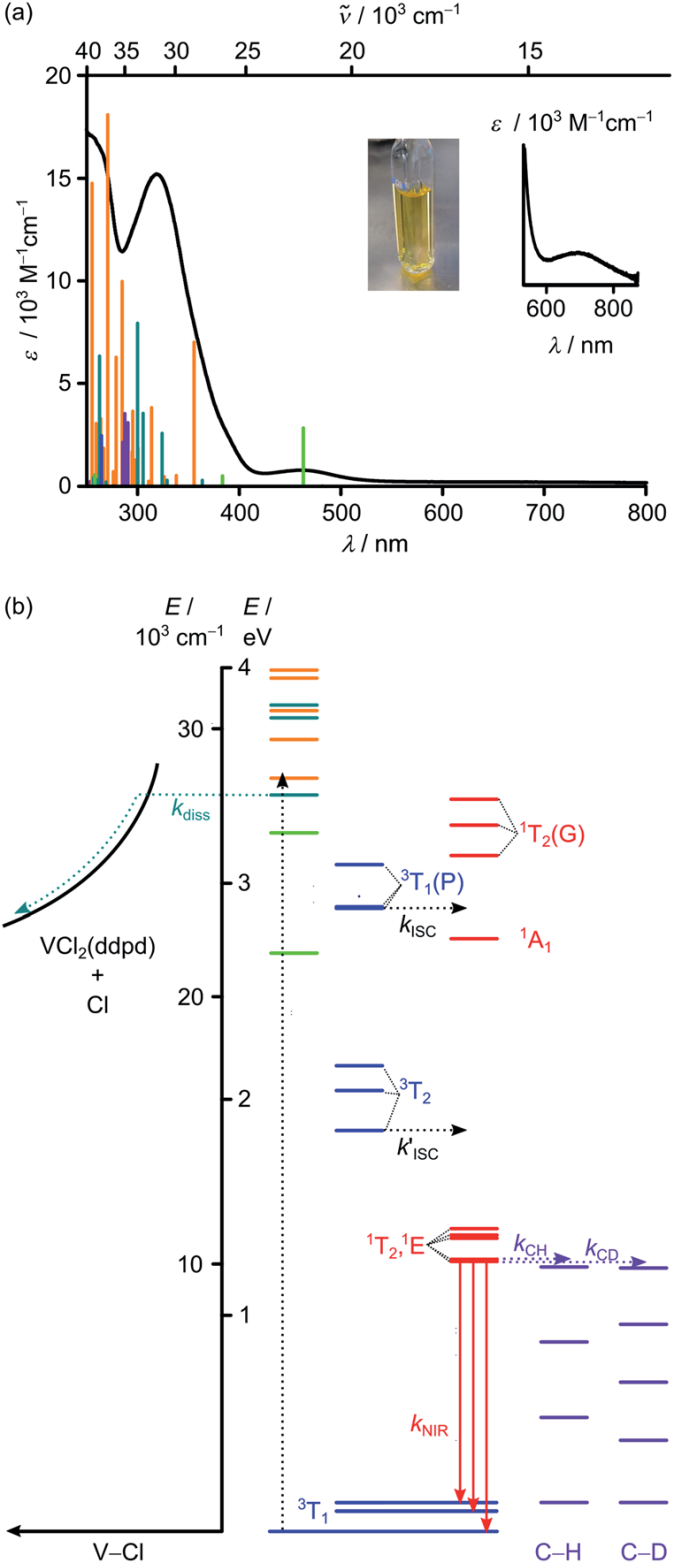
(a) Absorption spectrum including a zoom into the red spectral region (black) and photograph of VCl_3_(ddpd-[D_0_]) in CH_3_CN. The vertical bars correspond to spin-allowed CT transitions calculated by TDDFT-UKS (hypsochromically shifted by 3400 cm^−1^). The colour code of the vertical bars indicates the major character of the transition as ddpd → V ^3^LMCT (light green), Cl → V ^3^LMCT (petrol green), ILCT (orange) and V → ddpd ^3^MLCT (purple). (b) Jablonski diagram constructed from TDDFT-UKS (ddpd → V ^3^LMCT (light green), Cl → V ^3^LMCT (petrol green) and ILCT (orange)) and CASSCF-NEVPT2 calculations (^3^MC states in blue; ^1^MC states in red). Notation of MC states is according to octahedral symmetry. ISC = intersystem crossing; diss = V–Cl homolysis; NIR = NIR emission; C–H and C–D overtones in purple = multiphonon relaxation (experimental overtone energies from ref. 34).

The electronic absorption spectrum of VCl_3_(ddpd-[D_0_]) in acetonitrile reveals prominent absorption bands around 320 and 463 nm (31 250 and 21 598 cm^−1^) as well as a very weak band at 702 nm (14 245 cm^−1^; *ε* < 100 M^−1^ cm^−1^; Fig. 1a). TDDFT calculations on the geometry optimised triplet ground state (CPCM(acetonitrile)-RIJCOSX-UB3LYP-D3BJ-ZORA/def2-TZVPP; for details see Experimental section) were performed to assign the optical transitions (Fig. 1a, S1, S2 and Tables S1–S4, ESI‡). To better match the experiment the calculated transition energies were shifted hypsochromically by 3400 cm^−1^ (Fig. 1a). Partitioning of the density-matrix into ddpd, Cl and V fragments for charge transfer number analysis using the TheoDore package^66,67^ indicates the major character of the transitions as ddpd → V ^3^LMCT (light green), Cl → V ^3^LMCT (petrol green), ILCT (orange) and V → ddpd ^3^MLCT (purple) (Fig. 1a). CASSCF calculations reported in the accompanying paper^63^ suggest some double excitation character of the ILCT transitions which might well lower the energy of these states. The very weak low-energy band at 702 nm (*ε* < 100 M^−1^ cm^−1^) corresponds to metal-centred (MC) transitions from t_2g_ to e_g_ orbitals (^3^T_2_).

To better describe the split ^3^T_1_ ground state and the intra- and interconfigurational metal-centred transitions of the vanadium(iii) ion, we resorted to CASSCF(6,12)-FIC-NEVPT2 calculations on the CPCM(acetonitrile)-RIJCOSX-UB3LYP-D3BJ-ZORA/def2-TZVPP optimised triplet ground state geometry (for details see Experimental section). The active space included five 3d and five 4d orbitals as well as two occupied V–N/Cl σ-bonding orbitals occupied with six electrons (Table S5 and Fig. S3, ESI‡).^68^ Without including the dynamic correlation with NEVPT2, the ^1^E/^1^T_2_ and ^3^T_2_ states are quite close in energy (Table S6, ESI‡). Inclusion of the dynamic correlation with NEVPT2 lowers the energy of the ^1^E/^1^T_2_ states by *ca.* 2550 cm^−1^ and raises the energy of the ^3^T_2_ states by *ca.* 1200 cm^−1^. On this level of theory, the energy gap between the lowest singlet and triplet excited states amounts to *ca.* 3750 cm^−1^ (Fig. 1b). These calculations place the lowest excited triplet states of ^3^T_2_ parentage at 14 968/16 464/17 393 cm^−1^ above the split ^3^T_1_ ground state (0/755/1076 cm^−1^) (Fig. 1b). The lowest energy spin-allowed transition is calculated at 14 968 cm^−1^ in reasonable agreement with the experimental band maximum (14 245 cm^−1^, Fig. 1a inset). The calculated splitting of all ^3^T states is substantial reflecting the low symmetry of the complex. The five lowest excited singlet states of ^1^E/^1^T_2_ parentage are calculated at 10 086/10 161/10 949/11 068/11 309 cm^−1^. These spread over 1200 cm^−1^ (Fig. 1b). As the energy of the lowest singlet state is significantly lower than the lowest excited triplet state by *ca.* 4900 cm^−1^, NIR phosphorescence from the singlet state to the split ground state is conceivable (Fig. 1b). Extended calculations using an even larger active space will be presented in the accompanying paper.^63^

**Fig. 2:**
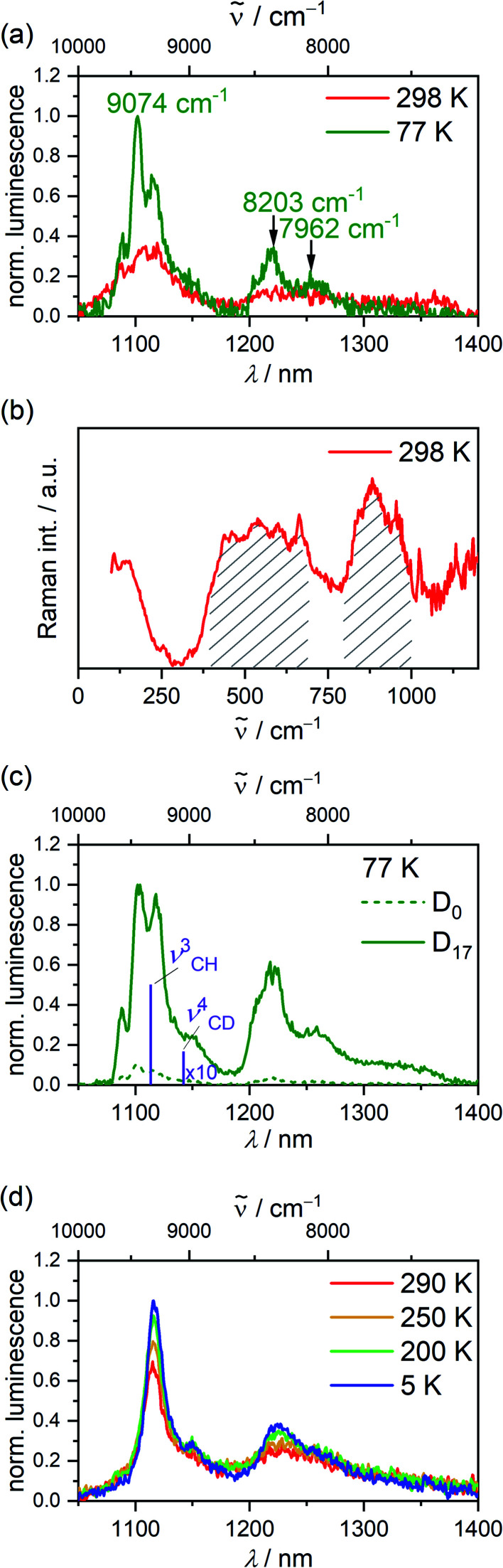
(a) Luminescence spectra of VCl_3_(ddpd-[D_0_]) at 298 K (red) and 77 K (green) (*λ*_exc_ = 350 nm), (b) solid state Raman spectrum of neat VCl_3_(ddpd-[D_0_]) at 298 K (*λ*_exc_ = 785 nm). Electronic Raman transitions between the ^3^T_1_ ground state components are highlighted. (c) Luminescence spectra of neat VCl_3_(ddpd-[D_0_]) (green dotted line) and VCl_3_(ddpd-[D_17_]) (green solid line) at 77 K (*λ*_exc_ = 350 nm). Purple bars indicate the positions of the ν^3^_CH_ and ν^4^_CD_ overtones. (d) Luminescence spectra of VCl_3_(ddpd-[D_17_]) as KBr pellet at 5–290 K (*λ*_exc_ = 350 nm).

Excitation of solid VCl_3_(ddpd-[D_0_]) with 350 nm (ILCT) at 298 K results in the appearance of two NIR emission bands (Fig. 2a). At 77 K, the emission bands increase in intensity and develop a characteristic fine structure (Fig. 2a). Discernible peaks occur at 1102, 1219 and 1256 nm (9074, 8203, 7962 cm^−1^). Considering the calculated ground state splitting, we assign these clearly visible band maxima to radiative transitions from the lowest excited singlet state(s) to the split ground state (Fig. 1b). The resulting experimental ground state splitting of *ca.* 800 and 1100 cm^−1^ excellently agrees with the CASSCF-NEVPT2 calculated splitting (755 and 1076 cm^−1^). The experimental ground state splitting refers to the geometry minimum of the singlet state, while the CASSCF-NEVPT2 calculated splitting refers to the ground state geometry. As spin–flip states are rather nested, the geometry differences should be marginal. Raman spectra of [V(H_2_O)_6_]^3+^ and [V(urea)_6_]^3+^ show broad electronic Raman transitions around 1900–2900 cm^−1^ and 1400 cm^−1^, respectively,^51,52^ due to the trigonal ground-state splitting. For VCl_3_(ddpd-[D_0_]), we observe two broad electronic Raman transitions around 500 and 900 cm^−1^ in its Raman spectrum in accordance with its lower symmetry (Fig. 2b). These energies fit well to the splitting assigned by luminescence spectroscopy (Fig. 2a) and obtained from the CASSCF-NEVPT2 calculations.

The finer details of the NIR luminescence band structure can be tentatively assigned to population of the close-lying singlet states with the difference between the two lowest singlet states calculated as 75 cm^−1^ and to enabling vibrations around 120 cm^−1^. Indeed, Cl–V–Cl deformation vibrations (125, 136, 159 cm^−1^; unscaled) appear in this energy region according to the DFT calculations (ESI, Fig. S4‡).

Upon pressurizing solid VCl_3_(ddpd-[D_0_]) to 7 kbar, two very weak NIR emission bands at approximately 9100 and 9280 cm^−1^ (lowest energy detectable with the employed detector) shift to higher energy by ≈10 cm^−1^ kbar^−1^. (Fig. S5, ESI‡). This hypsochromic shift of the emission bands differs from the bathochromic behaviour encountered by the d^3^ complex [Cr(ddpd)_2_]^3+^ at increasing pressure.^39^ This unusual pressure-sensitivity is probably a combined effect of energy changes of the emissive singlet states and of the ground state splitting under increasing pressure. The broad, electronic Raman bands assigned to transitions within the split ^3^T_1g_ ground state experience variations in intensity and broaden strongly with increasing pressure (Fig. S6, ESI‡). At pressures higher than 30 kbar the broadening, most likely due to effects of non-hydrostatic pressure, dominates and the bands can no longer be observed. These observations illustrate that luminescence shifts different from those for spin–flip transitions with nondegenerate ground states are expected for vanadium(iii) complexes.

The NIR luminescence of VCl_3_(ddpd-[D_17_]) at 298 K in the solid state under inert conditions (*λ*_exc_ = 350 nm) decays monoexponentially with *τ*^H^_298_ = 0.5 μs (ESI, Fig. S7‡). This long lifetime confirms the assignment as phosphorescence. With the fully deuterated ligand ddpd-[D_17_]^36^ installed in VCl_3_(ddpd-[D_17_]), the NIR luminescence intensity strongly increases compared to that of the non-deuterated complex (Fig. 2c). Concomitantly, the luminescence lifetime at 298 K increases to *τ*^D^_298_ = 3.3 μs (*λ*_obs_ = 1106 nm) and 3.4 μs (*λ*_obs_ = 1222 nm) (Fig. S8 and S9, ESI‡). The deuteration effect confirms that multiphonon relaxation (Fig. 1b) is substantial in VCl_3_(ddpd-[D_0_]). The estimated spectral overlap integral (SOI) of the second C–H_ar_ overtone *ν*^3^_CH_(ref. 34) of the ligand at 8972 cm^−1^ is significant, while the relevant third CD_ar_ overtone *ν*^4^_CD_ (ref. 34) at 8755 cm^−1^ has a much lower SOI due to its lower extinction coefficient (ESI, Fig. S10–S12‡). Based on the vibrational overtone analysis and SOI calculation, the rate constant for this overtone-mediated non-radiative decay mechanism should diminish by a factor of 36 (ESI, Fig. S10–S16‡). This qualitatively matches the observed intensity enhancement upon deuteration. The observation of an isotope effect confirms that multiphonon relaxation is a major non-radiative decay path of the singlet states in this complex dominating other non-radiative decays. This finding contrasts the observations for the homoleptic complex [V(ddpd)_2_][PF_6_]_3_ and its deuterated isotopologue where other decay pathways appear to dominate the non-radiative decay of the NIR-emissive states.^36^

Cooling solid VCl_3_(ddpd-[D_*n*_]) both as neat powder and as KBr pellet increases the luminescence intensity (Fig. 2a, d; and S17–S19, ESI‡). For example, cooling VCl_3_(ddpd-[D_17_]) from 290 to 200 K yields a 1.5-fold increased integrated NIR intensity, while further cooling to 5 K only has a minor effect (Fig. 2d; and S17–S19, ESI‡). This suggests the presence of a thermally activated non-radiative pathway accessible at temperatures above 200 K.

**Fig. 3:**
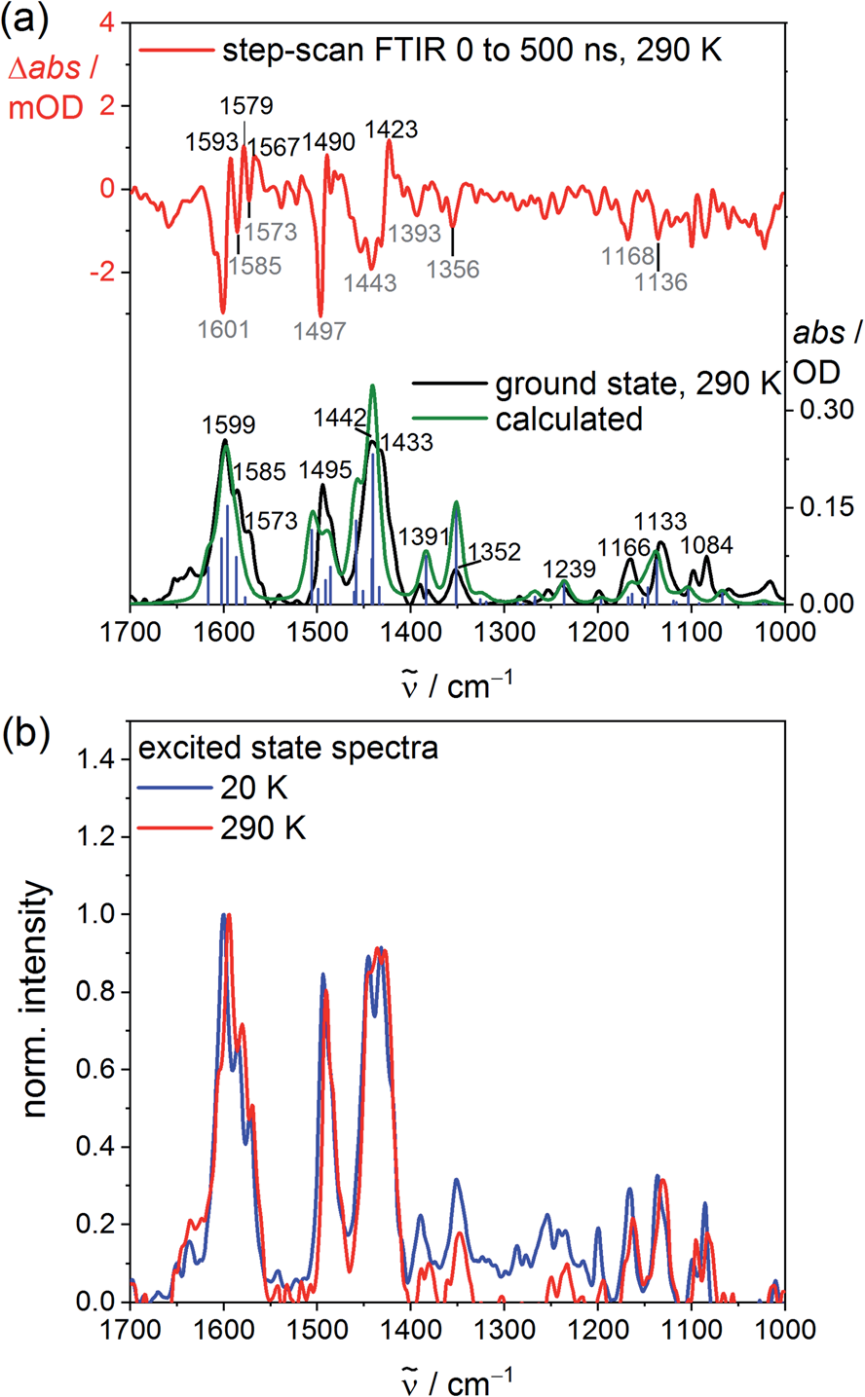
(a) Experimental (black), DFT-calculated ground state (green) and step-scan FTIR spectra (top, *λ*_exc_ = 355 nm; 0–500 ns) of VCl_3_(ddpd-[D_0_]) in a KBr pellet at 290 K (red) and (b) excited state FTIR spectra of VCl_3_(ddpd-[D_0_]) in a KBr pellet obtained from step-scan FTIR spectra (*λ*_exc_ = 355 nm; 0–500 ns) (small contributions of 3% of the respective ground state spectrum added to the step-scan spectrum) at 20 K (blue) and 290 K (red).

To probe the structure, the vibrational signature and possible distortions of the long-lived excited singlet states at high and low temperature, step-scan FTIR spectra of VCl_3_(ddpd-[D_0_]) and VCl_3_(ddpd-[D_17_]) were recorded in KBr pellets (Fig. 3; and S20–S23, ESI‡). The ground state FTIR spectra of VCl_3_(ddpd-[D_0_]) and VCl_3_(ddpd-[D_17_]) in KBr pellets at 290 K are well reproduced by DFT calculated frequencies scaled by 0.98 (Fig. 3a and S21, ESI‡). The characteristic *ν*_CC_, *ν*_CN_ and *δ*_CH_ modes of the terminal and central pyridine rings around 1599, 1495 and 1442 cm^−1^ shift to lower energy by approximately 35–75 cm^−1^ upon deuteration of the ligand in VCl_3_(ddpd-[D_17_]).

Step-scan FTIR spectra recorded for VCl_3_(ddpd-[D_0_]) and VCl_3_(ddpd-[D_17_]) in a KBr pellet with *λ*_exc_ = 355 nm collected over 0–500 ns at 290 and 20 K are depicted in Fig. 3a, S20, S21 and S23 (ESI‡). The long-lived excited singlet states give rise to positive and negative bands corresponding to the population of the excited singlet states and ground state bleach, respectively. The excited state spectra of VCl_3_(ddpd-[D_0_]) and VCl_3_(ddpd-[D_17_]) at 290 and 20 K are derived from the respective step-scan and the ground state spectra (Fig. 3b; and S22, ESI‡). Temperature has only a minor influence on the excited state spectra of VCl_3_(ddpd-[D_0_]) (Fig. 3b), but modifies the relative excited state IR intensities of the deuterated derivative VCl_3_(ddpd-[D_0_]) (Fig. S22, ESI‡).

The evolution of prominent IR bands after excitation of VCl_3_(ddpd-[D_0_]) over time was fitted in a global analysis giving a monoexponential decay with *τ*^H^_290K_ = 0.6 μs at 290 K (Fig. S24, ESI‡) excellently fitting to the decay observed by photoluminescence spectroscopy at 298 K. Cooling to 20 K approximately doubles the excited state lifetime to *τ*^H^_20K_ = 1.3 μs (Fig. S25, ESI‡). This confirms that thermally activated non-radiative pathways are operative at room temperature in addition to the multiphonon relaxation *via* C–H oscillators,^57^ which takes place at all temperatures. Surprisingly, the step-scan data of the deuterated complex VCl_3_(ddpd-[D_17_]) deliver excited state lifetimes of *τ*^D^_290K_ = 0.6 μs and *τ*^H^_20K_ = 1.2 μs at 290 and 20 K, respectively (Fig. S26 and S27, ESI‡), similar to VCl_3_(ddpd-[D_0_]). This differs from the higher room temperature lifetime of VCl_3_(ddpd-[D_17_]) *τ*^D^_298_ = 3.3/3.4 μs obtained by time-correlated single photon counting (Fig. S8 and S9, ESI;‡ see above). Possibly, the step-scan FTIR experiment mainly detects one of the emissive singlet states but fails to probe the second emissive singlet state. According to the CASSCF-NEVPT2 calculations, the two lowest singlet states derive from terms with essentially ^1^E and ^1^T_2_ character, respectively, with a very small energy difference of only 75 cm^−1^ (Table S6, ESI‡). As the orbital population of the ^1^T_2_-derived term matches that of the lowest term of the split ^3^T_2_ ground state (Fig. S3, ESI‡), this excited state possesses the same equilibrium nuclear configuration as the ground state (nested state). Consequently, step-scan FTIR spectroscopy would not be able to detect this excited state. Clearly, a model of the excited state decay of the two lowest energy singlet excited states to the split ground state requires at least five electronic states. For a kinetic model of the non-radiative decay *via* high- and low-frequency modes^57^ these comparably close-lying electronic states (Fig. 1b) would have to be combined with the different anharmonic vibrational C–H/C–D modes as well as the pyridine ring vibrational ladders. The latter modes are also affected by deuteration according to the ground state FTIR spectra of VCl_3_(ddpd-[D_0_]) and VCl_3_(ddpd-[D_17_]) (Fig. 3a and S21, ESI‡).

As VCl_3_(ddpd) is only poorly soluble in typical solvents, a detailed reliable study of its weak NIR luminescence in solution is unfortunately impeded, especially when exciting at the very weak ^3^MC band. Furthermore, we noted a follow-up reaction upon irradiating VCl_3_(ddpd-[D_0_]) at 350 ± 5 nm (ILCT) in acetonitrile solution. The absorption spectrum changes and an emission band at *ca.* 400 nm grows in over time (Fig. S28 and S29, ESI‡). The photostability is much higher under irradiation at 400 ± 5 nm including consideration for absorption and light intensity of the light source. (Fig. S30–S32, ESI‡). This suggests that the low energy ^3^T_2_, ^1^T_2_/^1^E and ddpd → V ^3^LMCT states are not responsible for the photoreactivity (Fig. 1b). At the higher excitation energy and with the assumption that LMCT states are likely involved (Fig. 1), we speculate that V–Cl homolysis could occur in solution. The well-known fact, that M–Cl bonds of reducible metal ions are prone to photoinduced homolysis has regained considerable interest in organic photoredox catalysis in particular for copper^69–71^ and nickel.^72–75^ VCl_3_ itself is photoreduced to vanadium(ii) in alcoholic solutions *via* excitation into LMCT states (chloride-to-vanadium or alkoxide-to-vanadium charge transfer).^76^ A ^3^LMCT state with chloride-to-vanadium character was found by TDDFT at 324 nm (state 14 shifted hypsochromically by 3400 cm^−1^, Tables S3, S4, and Fig. S2, ESI‡). This ^3^LMCT state could qualify as excited state with V–Cl dissociative character. To probe the conceivable V^III/II^ reduction process, a cyclic voltammogram of VCl_3_(ddpd-[D_0_]) was recorded in CH_3_CN. The cathodic scan reveals an irreversible reduction wave at *E*_p_ = −1.11 V *versus* ferrocene/ferrocenium with an oxidative follow-up wave at *E*_p_ = −0.25 V and a reductive wave at −0.83 V (Fig. S33, ESI‡). This behaviour can be associated with chloride loss upon electron capture, similar to the reported preparation of VCl_2_(py)_4_ from VCl_3_, pyridine and zinc dust as reductant.^77^ Consequently, we consider V–Cl bond homolysis as a viable reaction path under UV light photolysis in fluid solution. In contrast to this photoreactivity of the chlorido complex, the homoleptic complex [V(ddpd)_2_][PF_6_]_3_ appears comparably photostable in solution, which can be ascribed to the absence of suitable dissociative LMCT states.^36^

**Fig. 4:**
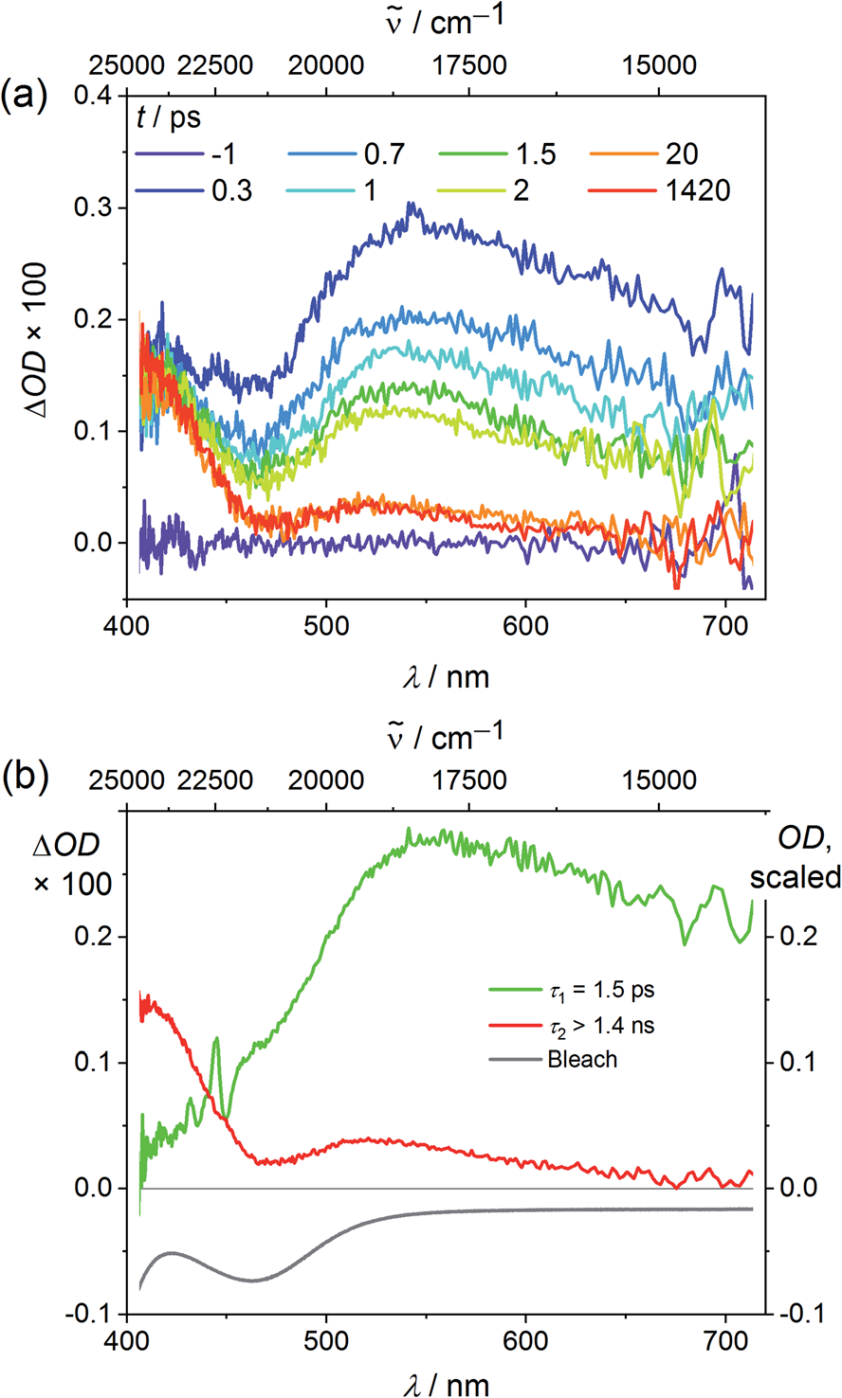
(a) Transient absorption spectra of VCl_3_(ddpd-[D_0_]) in CH_3_CN excited with fs laser pulses at 400 nm (298 K) and (b) decay associated amplitude spectra (red, green) with indicated lifetimes and the ground state bleach (grey).

Finally we explored the reaction path from the Franck–Condon excited triplet state to the long-lived singlet states by ultrafast transient absorption spectroscopy in CH_3_CN. To diminish the dissociative processes assigned to high-energy ^3^LMCT states with Cl → V character, 400 nm pulses were employed populating essentially ^3^LMCT states with NMe(ddpd) → V character (Tables S3, S4 and Fig. S2, ESI‡). After excitation, a broad excited state absorption (ESA) from 470–700 nm appears in addition to an ESA around 410 nm (Fig. 4a). The ground state bleach fits to the dip in the transient absorption spectrum around 463 nm (Fig. 1a and 4b).

The broad ESA evolves with *τ*_1_ = 1.5 ps to a long-lived state with a maximum at 543 nm (Fig. 4b). This state persists much longer than the time window of 1.4 ns of the pump-probe experiment, which is given by the length of the motorized delay stage in the setup. Since electronic relaxation between electronically excited states of the same spin multiplicity is typically rather fast, one of the lowest excited states of the different spin multiplicities should be responsible for the long-lived state, *i.e.* the ^3^T_2_ state or the ^1^E/^1^T_2_ states (Fig. 1). Significant population in a long living electronically excited triplet state should result in fluorescence, since the radiative transition to the ground state would be spin allowed and Laporte's rule is relaxed in the complex. However, no luminescence is observed at wavelengths below 1050 nm as would be expected for triplet states (Fig. 1). This excludes that a significant excited state population is trapped in any triplet state. A partitioning of excited state population in triplet and singlet states, as it was observed in [V(ddpd)_2_]^3+^ (ref. 36) does not occur with VCl_3_(dppd). The persistent TA component is therefore assigned to the long-lived ^1^E/^1^T_2_ states. Clearly, ISC to the singlet manifold and vibrational cooling proceed to completion within a few ps. The rate constant for ISC *k*_ISC_ is at least 1/*τ*_1_ = 6.7 × 10^11^ s^−1^. Trajectory surface hopping simulations within a linear vibronic coupling model will derive a detailed kinetic model of the initial dynamics and the efficiency of the ISC processes (*τ*_ISC,simulation_ = 1.7 ± 0.3 ps) in the accompanying paper.^63^

## Experimental

**Synthesis**. VCl_3_(ddpd-D_0_) was prepared according to ref. 58. The deuterated complex VCl_3_(ddpd-[D_17_]) was prepared analogously using the deuterated ligand ddpd-[D_17_] prepared according to ref. 36.

**Electrochemical experiments** were carried out on a BioLogic SP-200 voltammetric analyser using platinum wires as counter and working electrodes and a 0.01 M Ag/Ag[NO_3_] electrode as reference electrode. Cyclic voltammetry and square wave measurements were carried out at scan rates of 50–200 mV s^−1^ using 0.1 M [^*n*^Bu_4_N][PF_6_] in CH_3_CN as supporting electrolyte. Potentials are referenced against the ferrocene/ferrocenium couple.

**Photolysis experiments** were carried out in CH_3_CN using an Asahi Spectra Max-303 Xenon Light Source (300 W, Fig. S32, ESI‡), together with 350 ± 5 nm and 400 ± 5 nm filters, respectively.

**UV/Vis photoluminescence spectra during photolysis experiments** were collected on a Varian Cary Eclipse spectrometer.

**UV/Vis/NIR spectra** were recorded on a Varian Cary 5000 spectrometer using 1.0 cm cells.

**Raman and luminescence spectra under pressure** at wavelengths up to 1050 nm were measured with a Renishaw InVia microscope (488 and 785 nm laser wavelengths) and a HPDO diamond anvil cell.

**Temperature-dependent steady-state NIR luminescence experiments** down to 5 K were conducted on a Horiba Jobin Yvon Fluorolog 3-22 *τ* spectrometer equipped with a 450 W xenon lamp and a DSS – IGA020L NIR detector (850 nm < *λ*_em_ < 1550 nm). Spectral selection was realized with double and single grating monochromators in the excitation and emission paths, respectively (excitation: 1200 grooves per mm; near-IR emission 600 grooves per mm). A combination of two long-pass filters (FELH0500 Thorlabs, transmission ≥92% above 500 nm and FELH0850 Thorlabs, transmission ≥98% above 1000 nm) was used in the emission channel to avoid higher order excitation light. For the preparation of KBr pellets, the compounds (*ca.* 1.0 mg for VCl_3_(ddpd-[D_0_]) and 0.5 mg for VCl_3_(ddpd-[D_17_])) were mixed with dry KBr (*ca.* 200 mg, stored in a compartment dryer at 80 °C, purchased from Merck) and ground to a homogenous mixture. This mixture was filled into an evacuable pellet die with a diameter of 13 mm and sintered at a pressure of 0.75 GPa. Measurements on neat powders were conducted by homogenous spreading of the neat sample between two CaF_2_ windows (13 mm diameter, 1 mm thick). Experiments at temperatures between 5 K and 290 K were performed using a closed-cycle helium cryostat (ColdEdge, 101J cryocooler). The cryocooler was equipped with a pellet holder (copper) and CaF_2_ windows.

**Steady-state NIR luminescence experiments** on neat samples down to 77 K were conducted on a Horiba Fluorolog-3 spectrofluorimeter equipped with a 450 W xenon lamp for steady-state measurements. Emitted light was detected either by a Hamamatsu R2658P PMT detector (200 nm < *λ*_em_ < 1010 nm) or by a Hamamatsu H10330-75 PMT detector (950 nm < *λ*_em_ < 1700 nm). Spectral selection in the excitation path was accomplished by a DFX monochromator (double gratings: 1200 grooves per mm, 330 nm blaze) and in the emission paths in the visible/NIR spectral region (*λ*_em_ < 1010 nm) by a spectrograph iHR550 (single gratings: either 1200 grooves per mm, 500 nm blaze or 950 grooves per mm, 900 nm blaze) and in the NIR spectral region (*λ*_em_ > 950 nm) by a spectrograph iHR320 (single grating: 600 grooves per mm, 1000 nm blaze).

**Near-IR luminescence lifetimes** of the phosphorescent transitions were determined at 298 K (solid samples in standard NMR tubes under argon) with a PTI Quantamaster QM4 spectrofluorimeter equipped with a 75 W continuous xenon short arc lamp as excitation source (Hamamatsu L4633: pulse width *ca.* 1.5 μs FWHM). Emission was monitored using a PTI P1.7R detector module (Hamamatsu PMT R5509-72 with a Hamamatsu C9525 power supply operated at 1500 V and a Hamamatsu liquid N_2_ cooling unit C9940 set to −80 °C). For the measurements above 1000 nm, a long-pass filter RG-850 (Schott, 3.0 mm thickness, transmission >98% above 970 nm) was used in the emission channel in order to avoid higher order excitation light. Spectral selection was achieved by single grating monochromators (excitation: 1200 grooves per mm, 300 nm blaze; Vis emission: 1200 grooves per mm, 500 nm blaze; near-IR emission: 600 grooves per mm, 1200 nm blaze) and an additional UG11 bandpass filter (Schott, 3.0 mm thickness) in the excitation channel. Lifetime data analysis (deconvolution, statistical parameters, *etc.*) was performed using the software package FeliX32 from PTI. Lifetimes were determined by deconvolution of the decay profiles with the instrument response function, which was determined using an empty NMR tube as scatterer. Estimated uncertainties for the lifetimes of the near-IR emission determined with this setup are 20%.

**Time-resolved FTIR experiments** were performed with the FTIR spectrometer Bruker Vertex 80v, operated in the step-scan mode. A liquid-nitrogen-cooled mercury cadmium telluride (MCT) detector (Kolmar Tech., Model KV100-1-B-7/190) with a rise time of 25 ns, connected to a fast preamplifier and a 14 bit transient recorder board (Spectrum Germany, M3I4142, 400 MS s^−1^), was used for signal detection and processing. The laser setup used for the measurements includes a Q-switched Nd:YAG laser (Innolas SpitLight Evo I) generating pulses with a pulse duration of about 6 ns at a repetition rate of 100 Hz. The third harmonic (355 nm) of the Nd:YAG laser was used directly for sample excitation. The UV pump beam was attenuated to about 1.8 mJ per shot at a diameter of 9 mm. The beam was directed onto the sample and adjusted to have a maximal overlap with the IR beam of the spectrometer. The sample chamber was equipped with anti-reflection-coated germanium filters to prevent the entrance of laser radiation into the detector and interferometer compartments. The KBr pellets were prepared as described in the section on luminescence spectroscopy, however, with a smaller amount of sample of *ca.* 0.2 mg and *ca.* 200 mg KBr. The strongest peak in the ground state spectrum showed an absorption of about 0.4 OD with the mentioned concentration. *T*-dependent measurements were performed using a closed-cycle helium cryostat (ARS Model DE-202A) reaching a temperature of about 20 K at the sample. The cryocooler was equipped with a pellet holder and CaF_2_ windows. The temporal resolution of the 14 bit transient recorder board was chosen to 5 ns for VCl_3_(ddpd-[D_0_]) and 10 ns for VCl_3_(ddpd-[D_17_]). The time where the laser pulse reached the sample was set as zero point in all spectra. The time delay between the start of the experiment and the laser pulse was controlled with a Stanford Research Systems DG535 delay generator. The spectral region was limited by undersampling to 1975 to 0 cm^−1^ for VCl_3_(ddpd-[D_0_]) and 988–1975 cm^−1^ for VCl_3_(ddpd-[D_17_]) with a spectral resolution of 4 cm^−1^ resulting in 1110 and 555 interferogram points, respectively. An IR broadband filter (850–1750 cm^−1^) and CaF_2_ windows (no IR transmission <1000 cm^−1^) prevented problems when performing a Fourier transformation (*i.e.* no IR intensity outside the measured region should be observed). FTIR ground state spectra were recorded systematically to check if there is no sample degradation. Estimated uncertainties for the excited state lifetimes are on the order of 10%.

**Transient absorption spectra** of VCl_3_(ddpd-[D_0_]) were recorded applying a pump-probe setup with an excitation wavelength of 400 nm. The setup is pumped by a Ti:sapphire laser system (Spectra-Physics, Spitfire Pro) which provides ultrashort laser pulses centred at 800 nm with a repetition rate of 1 kHz. By frequency doubling its output with a BBO-crystal pump pulses with a centre wavelength of 400 nm and a pulse duration of 200 fs were obtained. For probing, a white light continuum generated with a CaF_2_ crystal was used. Both beams, with polarizations arranged in magic angle, were focused onto the sample leading to pump and probe spots with diameters of 170 μm and 80 μm, respectively. Transient absorption spectra were recorded by dispersing the probe beam after the sample with a prism and detecting its spectral intensity distribution with a CCD array. The metal complex was dissolved in acetonitrile under argon atmosphere and the obtained sample was filled into a 1 mm fused silica cuvette. The concentration was 1.5 × 10^−3^ M resulting in an optical density of 0.18 at 400 nm. Significantly higher concentrations were not accessible because of the moderate solubility of the compound in acetonitrile.

**Quantum chemical calculations**. The characterization of the absorption spectrum was done employing two types of quantum chemical calculations: (i) density functional theory in its unrestricted form and (ii) multiconfigurational theory with an active space tailored to predict the MC states. The first method is labelled as “Unrestricted Kohn–Sham” orbitals DFT (UKS), the second as “SOC-CASSCF(6,12)-FIC-NEVPT2”. These two methods are complementary to each other, as the first gives energies of the CT states, while the second one provides the energies of the MC states and the ground state splitting.^78^

Unrestricted Kohn–Sham orbitals DFT (UKS): All calculations were performed using the quantum computing suite ORCA 4.0.1.12.^79^ Geometry optimization (Tables S1 and S2‡) was performed using unrestricted Kohn–Sham orbitals DFT (UKS) and the B3LYP functional^80–82^ in combination with Ahlrichs' split-valence triple-*ζ* basis set def2-TZVPP for all atoms.^83,84^ Tight convergence criteria were chosen for DFT-UKS calculations (keywords tightscf and tightopt). All DFT-UKS calculations make use of the resolution of identity RIJ (Split-RI-J) approach for the Coulomb term in combination with the chain-of-spheres approximation for the exchange term (COSX).^85,86^ The zero order relativistic approximation was used to describe relativistic effects in all calculations (keyword ZORA).^87^ Grimme's empirical dispersion correction D3(BJ) was employed (keyword D3BJ).^88,89^ To account for solvent effects, a conductor-like screening model (keyword CPCM) modelling acetonitrile was used in all calculations.^90,91^ TDDFT-UKS calculations were performed at the same level of theory using unrestricted Kohn–Sham orbitals (UKS). Fifty vertical spin-allowed transitions were calculated (Tables S3 and S4‡).

Harmonic frequency calculations for the IR assignments were performed using Turbomole 7.4 (ref. 92 and 93) on the optimized geometry (RIJCOSX-UB3LYP-D3BJ/def2-TZVP). The vibrational frequencies were scaled by a factor of 0.98, which is typical for the chosen method and basis set, to minimize the differences between the experimental and calculated frequencies. A Gaussian convolution with a full-width at half-maximum of 15 cm^−1^ was applied to the calculated vibrational transitions.

SOC-CASSCF(6,12)-FIC-NEVPT2: calculations of ground- and excited-state properties with respect to metal-centered (MC) states were performed using the complete-active-space self-consistent field method in conjunction with the fully internally contracted N-electron valence perturbation theory to second order based on a fully internally contracted (FIC) wave function (FIC-NEVPT2)^94,95^ in order to recover missing dynamic electron correlation. In order to accurately model the ligand field, active spaces were chosen to encompass the dominating bonding/antibonding orbitals formed between vanadium and the ligand. An active space of (6,12) along with 10 triplet roots and 9 singlet roots was selected (Tables S5 and S6‡). In addition to the minimal active space of (2,5) comprising the 3d orbitals, two occupied V–N σ bonding orbitals and a second d shell^96^ were included in these calculations.

## Conclusions

The pseudo-octahedral vanadium(iii) complex VCl_3_(ddpd) with the strong-field ligand ddpd shows spin–flip phosphorescence at room temperature at 1102, 1219 and 1256 nm after excitation into charge-transfer bands. Several factors are relevant for this emission from a 3d transition metal complex to occur:

(i) The ligand field splitting in VCl_3_(ddpd) is large enough to place the emissive singlet states ^1^E/^1^T_2_ below the distorted metal-centred triplet excited states ^3^T_2_.

(ii) Lower temperature disables thermally activated non-radiative pathways increasing the photoluminescence, yet even at room temperature a weak emission is still observed.

(iii) Deuteration of the ddpd ligand reduces non-radiative energy transfer to C–H overtones increasing the photoluminescence.

(iv) The radiative rate might be higher in non-centrosymmetric pseudo-octahedral vanadium complexes, although this effect of Laporte's parity rule was not experimentally confirmed in this particular case.

(v) The ISC rate constant from the triplet to the singlet manifold is high (*k*_ISC_ > 6.7 × 10^11^ s^−1^) as confirmed by the molecular dynamics simulations in the accompanying paper.^63^ This high rate could be an effect of the heavier chloride atoms (heavy atom effect), efficient vibronic coupling and/or enhanced SOC due to large differences in orbital type between the two states (^3^LMCT → ^1^MC),^97^ although other ultrafast pathways might still compete with ISC.

Challenges with the emission from excited states of d^2^-VCl_3_(ddpd) arise from the large ground state splitting which spreads the emission bands over *ca.* 2400 cm^−1^. This range is larger by almost two orders of magnitude than the corresponding spin–flip emission of d^3^-metal complexes with orbitally non-degenerate ground states, a very significant difference for transitions involving essentially nested states. The considerable ground state splitting further reduces the already small energy gap between the emissive state and the ground state enabling a higher non-radiative decay according to the energy gap law. A second aspect of VCl_3_(ddpd) as phosphorescent emitter concerns the excited state reactivity of LMCT states with chloride → vanadium charge-transfer character in solution. As these states can lead to V–Cl homolysis in solution reducing the photoluminescence and finally decomposing the complex, solution photostability is a particularly important aspect for future applications of vanadium(iii) complexes in solution.

This study emphasises that design strategies toward efficient d^2^-NIR emitters require a particular attention to the ISC efficiency from the triplets to the singlet states and potential dissociative unimolecular reactions at ultrafast timescales as well as on the radiative and non-radiative relaxation of the singlets at longer times. Details of the ultrafast excited state dynamics of VCl_3_(ddpd) up to 10 ps are discussed in the accompanying paper.^63^

## Data availability

Experimental and computational data are available as ESI.

## Author contributions

MD prepared the complex, performed all ground state characterization and photolysis experiments and the quantum chemical calculations, JK and MS measured and analysed the luminescence and lifetime data of the neat complex, measured and analysed the NIR absorption data and performed the SOI calculations, CD and CR measured and analysed the Raman spectra and the spectra under pressure, PB, GNS and MG measured and analysed the step-scan FT-IR spectra and the temperature dependent luminescence spectra of the complex in KBr pellets, AK and SL measured and analysed the transient absorption spectra, MS and KH devised the concept. KH supervised the project and wrote the manuscript.

## Conflicts of interest

There are no conflicts to declare.

## Supplementary Material

SC-012-D1SC02137K-s001
