# Supplementary material for: Ultrafast and long-time excited state kinetics of an NIR-emissive vanadium(iii) complex I: synthesis, spectroscopy and static quantum chemistry
Source: Chem Sci. 2021 Jul 15;12(32):10780–90. doi: 10.1039/d1sc02137k (PMC8372323; doi:10.1039/d1sc02137k)
Supplement: SC-012-D1SC02137K-s001 [file SC-012-D1SC02137K-s001.pdf]

Electronic Supplementary Material (ESI) for Chemical Science.
This journal is © The Royal Society of Chemistry 2021

Ultrafast and long-time excited state kinetics of an NIR-emissive vanadium(III) complex I: Synthesis, spectroscopy and static quantum chemistry†

Matthias Dorn[a], Jens Kalmbach[b], Pit Boden[c], Ayla Kruse[d], Chahinez Dab[e], Christian Reber[e], Gereon Niedner-Schatteburg[c], Stefan Lochbrunner[d], Markus Gerhards[c], Michael Seitz*[b], Katja Heinze*[a]

a. Department of Chemistry, Johannes Gutenberg University of Mainz, Duesbergweg 10-14, 55128 Mainz, Germany.

b. Institute of Inorganic Chemistry, University of Tübingen, Auf der Morgenstelle 18, 72076 Tübingen, Germany.

c. Department of Chemistry and Research Center Optimas, Technical University Kaiserslautern, Erwin-Schrödinger-Straße, 67663 Kaiserslautern, Germany.

d. Institute for Physics and Department of Life, Light and Matter, University of Rostock, 18051 Rostock, Germany.

e. Département de chimie, Université de Montréal, Montréal, Québec, H3C 3J7, Canada.

Figure S1. Geometry of VCl_3(ddpd) calculated by DFT-UKS [CPCM(acetonitrile)-RIJCOSX-B3LYP-D3BJ-ZORA/def2-TZVPP].

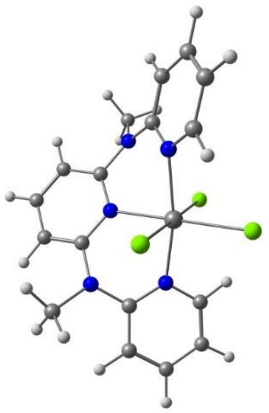

Figure S2. TD-DFT-UKS charge transfer (CT) numbers of VCl_3(ddpd) defined from 0 to 1 of the lowest-lying 50 triplet states.

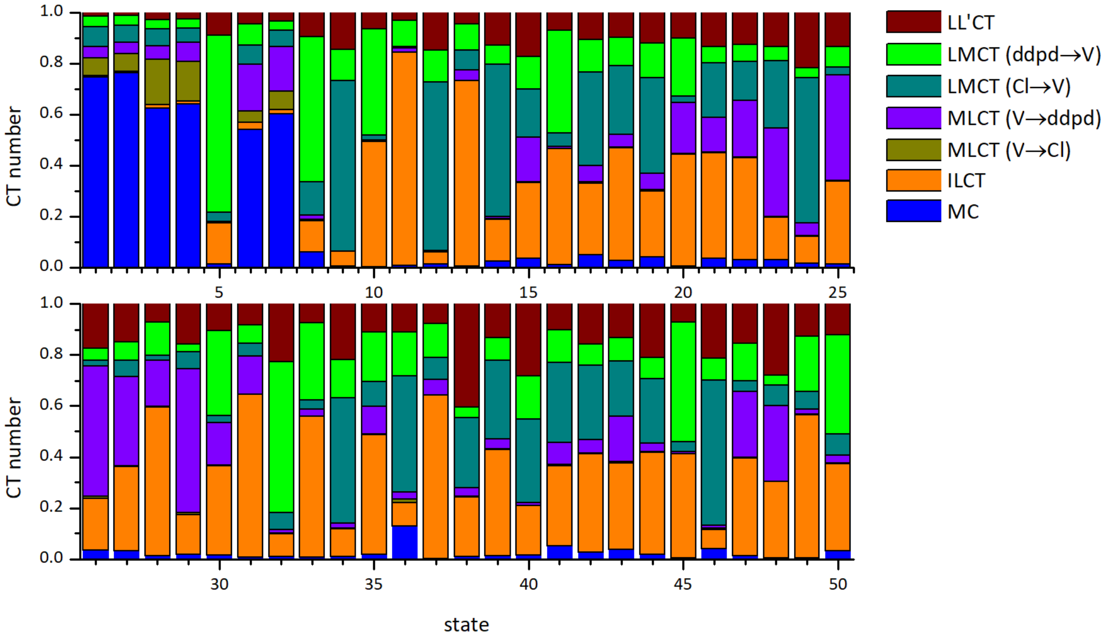

Table S1 Bond distances / Å and angles / ° of *mer*-VCl_3(ddpd)[S1] (XRD and DFT-UKS).

	V-N1/ V-N2/ V-N3	N1-V-N2/ N1-V-N3 N2-V-N3	V-Cl1 V-Cl2 V-Cl3	Cl1-V-Cl2 Cl1-V-Cl3 Cl2-V-Cl3	Shape parameter *S*(OC-6)
XRD	2.123(3)	84.68(12)	2.354(12)	90.42(4)	0.48
	2.119(3)	170.41(12)	2.325(13)	178.75(5)	
	2.109(3)	85.85(12)	2.335(12)	90.72(4)	
DFT-UKS	2.123	84.25	2.387	90.19	0.64
	2.114	168.53	2.375	179.66	
	2.123	84.28	2.386	90.08	

Table S2. Cartesian Coordinates of the DFT-UKS calculated ground state geometry of *mer*-VCl_3(ddpd).

atomic number	x	y	z
23	0.002946000	-0.007579000	0.104116000
7	0.004059000	-0.004113000	-2.010521000
7	-0.150783000	-2.114360000	-0.112225000
7	0.165427000	2.099835000	-0.104000000
6	-0.723824000	-0.918934000	-2.699791000
6	-0.737837000	-0.939401000	-4.091568000
6	0.000253000	-0.000559000	-4.786831000
6	0.740112000	0.936459000	-4.090917000
6	0.729983000	0.912705000	-2.699013000
1	-1.292363000	-1.701307000	-4.613717000
1	1.294128000	1.698627000	-4.613101000
6	0.410027000	-2.897831000	0.830558000
6	0.053717000	-4.212499000	1.014748000
6	-0.929873000	-4.745971000	0.182300000
6	-1.466786000	-3.972585000	-0.825731000
6	-1.029273000	-2.646721000	-0.976471000
1	-1.258101000	-5.768867000	0.305373000
1	1.139025000	-2.414357000	1.459686000
1	0.518604000	-4.795997000	1.794537000
1	-2.188579000	-4.392248000	-1.506323000
6	1.040352000	2.634690000	-0.970096000
6	1.477613000	3.960305000	-0.817174000
6	0.945395000	4.730647000	0.195745000
6	-0.033838000	4.194365000	1.031140000
6	-0.390784000	2.880151000	0.844004000
1	1.274108000	5.753222000	0.320160000
1	2.196282000	4.382386000	-1.499379000
1	-0.494924000	4.775114000	1.815197000
1	-1.116559000	2.394818000	1.475435000
7	-1.495888000	-1.869126000	-2.024261000
6	-2.778623000	-2.243367000	-2.633240000
1	-2.695669000	-3.084717000	-3.323231000
1	-3.470609000	-2.499142000	-1.835582000
1	-3.169494000	-1.382583000	-3.165664000
7	1.503910000	1.860572000	-2.022411000
6	2.781091000	2.242294000	-2.638419000
1	2.689775000	3.085259000	-3.325620000
1	3.477050000	2.499441000	-1.844655000
1	3.172865000	1.384873000	-3.175646000
1	-0.000860000	0.000776000	-5.868145000
17	-0.007942000	-0.016751000	2.479265000
17	-2.374490000	0.195152000	0.090701000
17	2.382361000	-0.201957000	0.106198000

Table S3. TD-DFT calculated spin-allowed transitions (UKS, unshifted). The assignment of the UKS states corresponds to the largest contribution to the charge transfer number (Fig. S2).

state	$\tilde{\nu}$ / cm^{-1}	λ / nm	E / eV	f_{osc}	assignment
1	2431	4114	0.301	7.24E-05	^{3}MC
2	3593	2783	0.445	4.10E-08	^{3}MC
3	17221	581	2.135	1.11E-05	^{3}MC
4	17358	576	2.152	6.54E-05	^{3}MC
5	18198	550	2.256	1.14E-02	^{3}LMCT(ddpd→V)
6	21692	461	2.689	1.35E-05	^{3}MC
7	22346	448	2.771	1.15E-04	^{3}MC
8	22681	441	2.812	2.01E-03	^{3}LMCT(ddpd→V)
9	24096	415	2.988	1.18E-03	^{3}LMCT(Cl→V)
10	24734	404	3.067	2.80E-02	^{3}LMCT(ddpd→V)
11	26178	382	3.246	2.11E-03	^{3}ILCT(ddpd)
12	26983	371	3.346	1.16E-03	^{3}LMCT(Cl→V)
13	27255	367	3.379	1.89E-03	^{3}ILCT(ddpd)
14	27465	364	3.405	1.03E-02	^{3}LMCT(Cl→V)
15	28474	351	3.530	1.54E-02	^{3}LMCT(Cl→V),^{3}ILCT (ddpd)
16	28777	348	3.568	9.71E-04	^{3}LMCT(ddpd→V), ^{3}ILCT (ddpd)
17	29334	341	3.637	1.42E-02	^{3}LMCT(ddpd,Cl→V)
18	29878	335	3.704	7.47E-03	^{3}LMCT(ddpd,Cl→V), ^{3}ILCT(ddpd)
19	29940	334	3.712	3.17E-02	^{3}LMCT(ddpd,Cl→V)
20	30211	331	3.746	5.13E-03	^{3}LMCT(ddpd→V),^{3}ILCT(ddpd)
21	30469	328	3.778	1.46E-02	^{3}ILCT(ddpd)
22	30562	327	3.789	6.74E-03	^{3}ILCT(ddpd)
23	30931	323	3.835	4.33E-04	^{3}LMCT(Cl→V)
24	30979	323	3.841	6.18E-05	^{3}LMCT(Cl→V)
25	31037	322	3.848	1.24E-02	^{3}MLCT(V→ddpd)
26	31397	319	3.893	1.42E-02	^{3}MLCT(V→ddpd)
27	31666	316	3.926	8.54E-03	^{3}MLCT(V→ddpd)
28	31726	315	3.934	3.99E-02	^{3}ILCT(ddpd)
29	32248	310	3.998	1.74E-04	^{3}MLCT(V→ddpd)
30	32468	308	4.025	2.51E-02	^{3}LMCT(ddpd→V)
31	32489	308	4.028	2.70E-03	^{3}ILCT(ddpd)
32	32765	305	4.062	3.27E-05	^{3}LMCT(ddpd→V)
33	32819	305	4.069	2.86E-03	^{3}LMCT(ddpd→V), ^{3}ILCT(ddpd)
34	33478	299	4.151	6.89E-06	^{3}LMCT(Cl→V)
35	33557	298	4.161	7.23E-02	^{3}ILCT(ddpd)
36	33795	296	4.190	8.51E-04	^{3}LMCT(Cl→V)
37	34118	293	4.230	7.47E-03	^{3}ILCT(ddpd)
38	34423	291	4.268	9.86E-03	^{3}LLCT(Cl→ddpd)
39	34530	290	4.281	1.32E-02	^{3}LMCT(ddpd,Cl→V), ^{3}ILCT(ddpd)
40	34674	288	4.299	2.53E-02	^{3}LMCT(ddpd,Cl→V)
41	34953	286	4.334	1.29E-03	^{3}LMCT(ddpd,Cl→V)
42	35026	286	4.343	4.10E-03	^{3}LMCT(ddpd,Cl→V), ^{3}ILCT(ddpd)
43	35063	285	4.347	7.91E-04	^{3}LMCT(ddpd,Cl→V), ^{3}ILCT(ddpd)
44	35162	284	4.360	1.23E-02	^{3}LMCT(ddpd,Cl→V), ^{3}ILCT(ddpd)
45	35386	283	4.387	2.20E-03	^{3}LMCT(ddpd→V)
46	35549	281	4.408	1.64E-03	^{3}LMCT(Cl→V)
47	35791	279	4.438	5.90E-02	^{3}ILCT(ddpd)
48	36062	277	4.471	9.19E-04	^{3}MLCT(V→ddpd)
49	36377	275	4.510	1.06E-05	^{3}ILCT(ddpd)
50	36670	273	4.547	4.40E-03	^{3}LMCT(ddpd→V)

Table S4. Difference electron densities of the 50 lowest-lying TD-DFT-UKS calculated states (isosurface value at 0.007 a.u.; purple = electron depletion; orange = electron gain; hydrogen atoms omitted).

#1
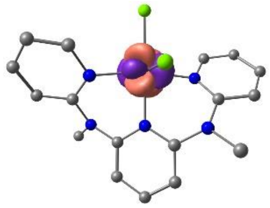
#2
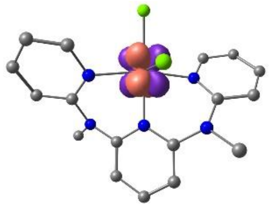
#3
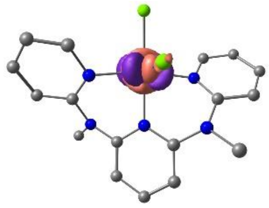
#4
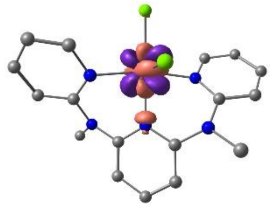
#5
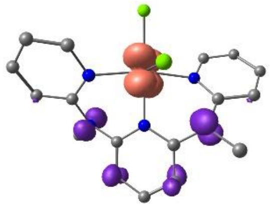
#6
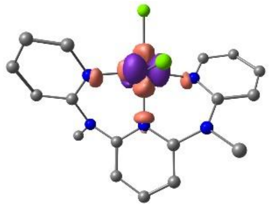
#7
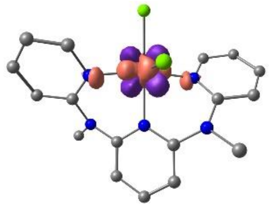
#8
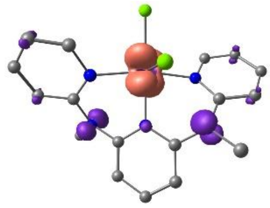
#9
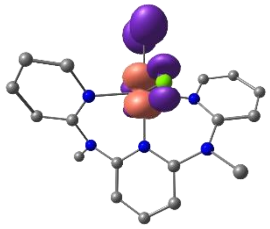
#10
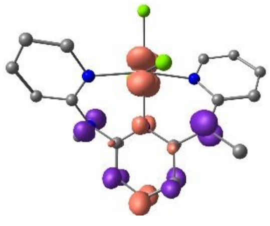
#11
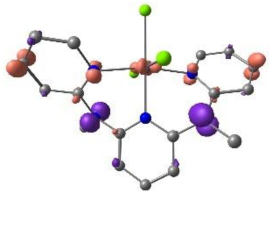
#12
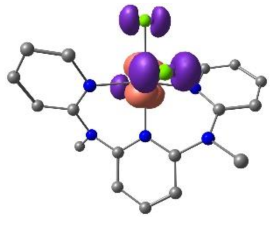
#13
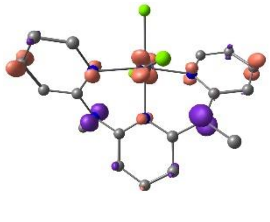
#14
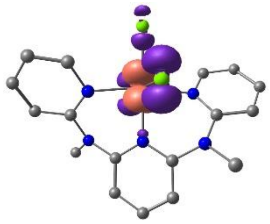
#15
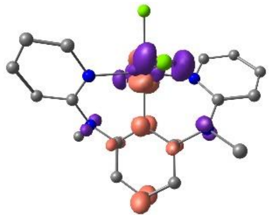
#16
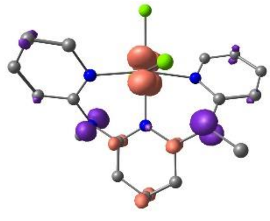
#17
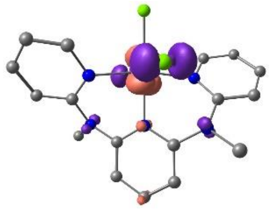
#18
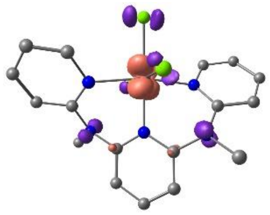
#19
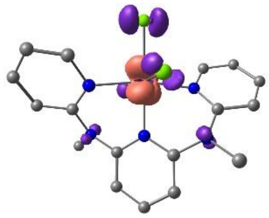
#20
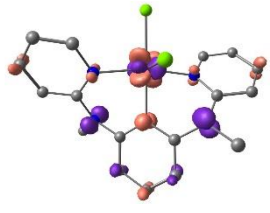
#21
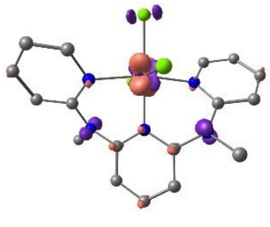
#22
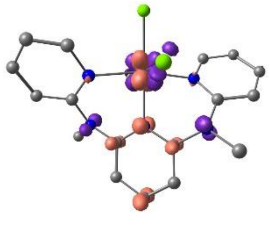
#23
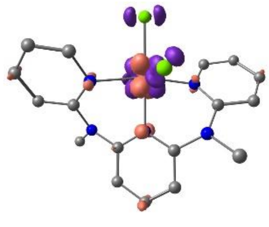
#24
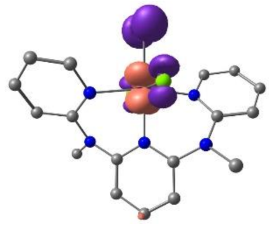
#25
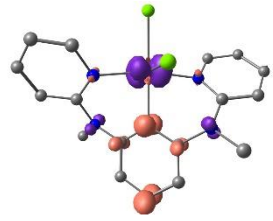
#26
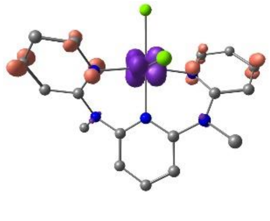
#27
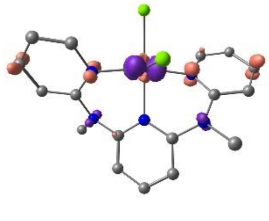
#28
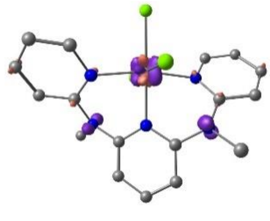

#29

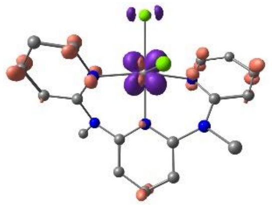

#30

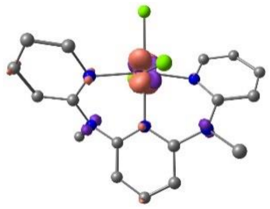

#31

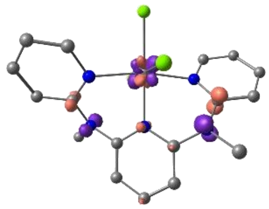

#32

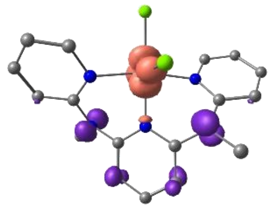

#33

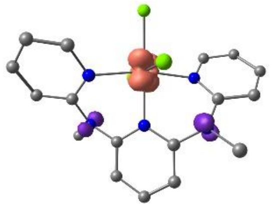

#34

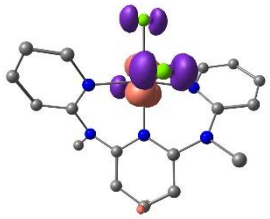

#35

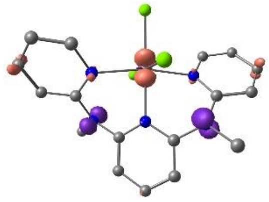

#36

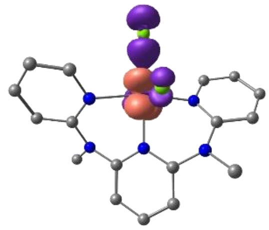

#37

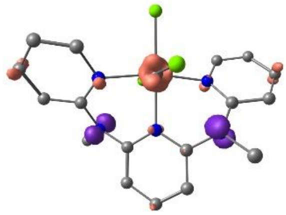

#38

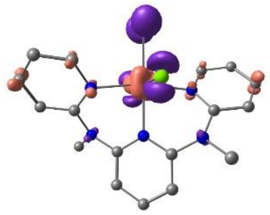

#39

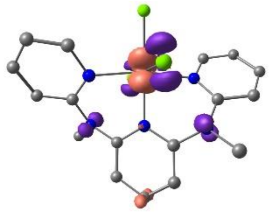

#40

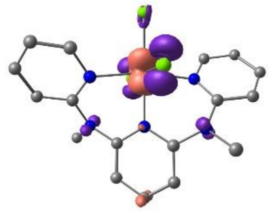

#41

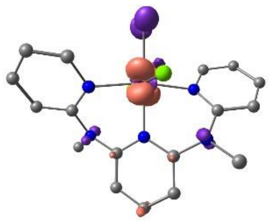

#42

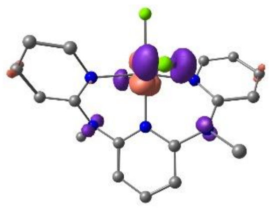

#43

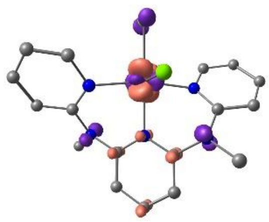

#44

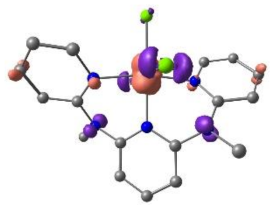

#45

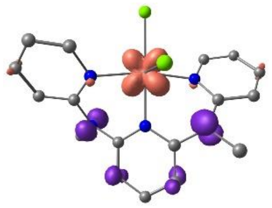

#46

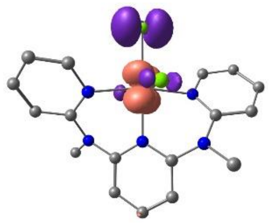

#47

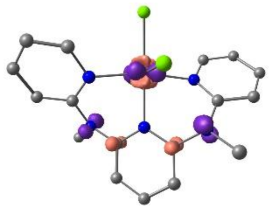

#48

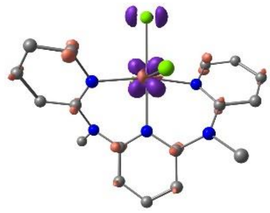

#49

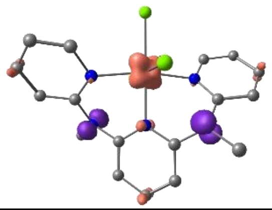

#50

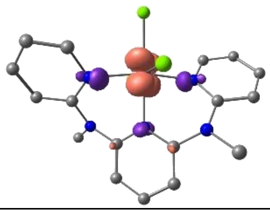

Table S5. Orbital energies/Hartree of the canonical orbitals used in the active space of the CASSCF(6,12)-FICNEVPT2 calculation, depicted at a contour value of 0.05 a.u. (hydrogen atoms omitted for clarity).

MO #	*E* / H	orbital	MO #	*E* / H	orbital
111	–0.53269	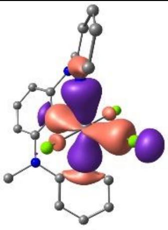	117	+0.15634	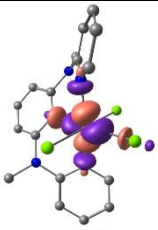
112	–0.48346	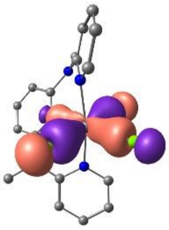	118	+0.87582	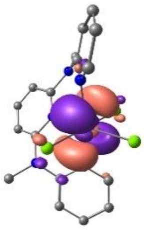
113	+0.02254	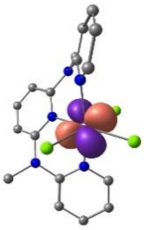	119	+0.90233	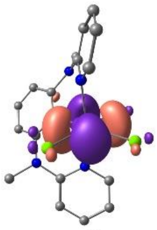
114	+0.02553	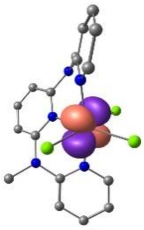	120	+0.91763	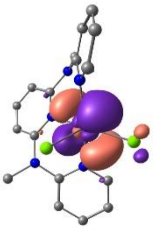
115	+0.02877	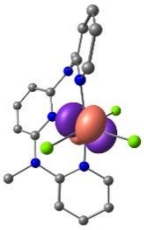	121	+1.13585	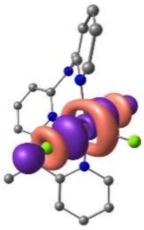
116	+0.10787	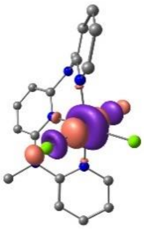	122	+1.27999	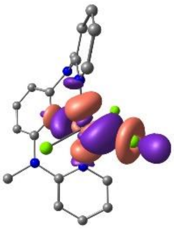

Figure S3. Energy diagram of the electronic states of VCl_3(ddpd) constructed from CASSCF(6,12)-FIC-NEVPT2 energies with spin densities in orange (0.007 a.u. isosurface value, hydrogen atoms omitted for clarity, triplet states in blue, singlet states in red; a coordinate system referring to the displayed structures and d orbital labels is shown in the grey box).

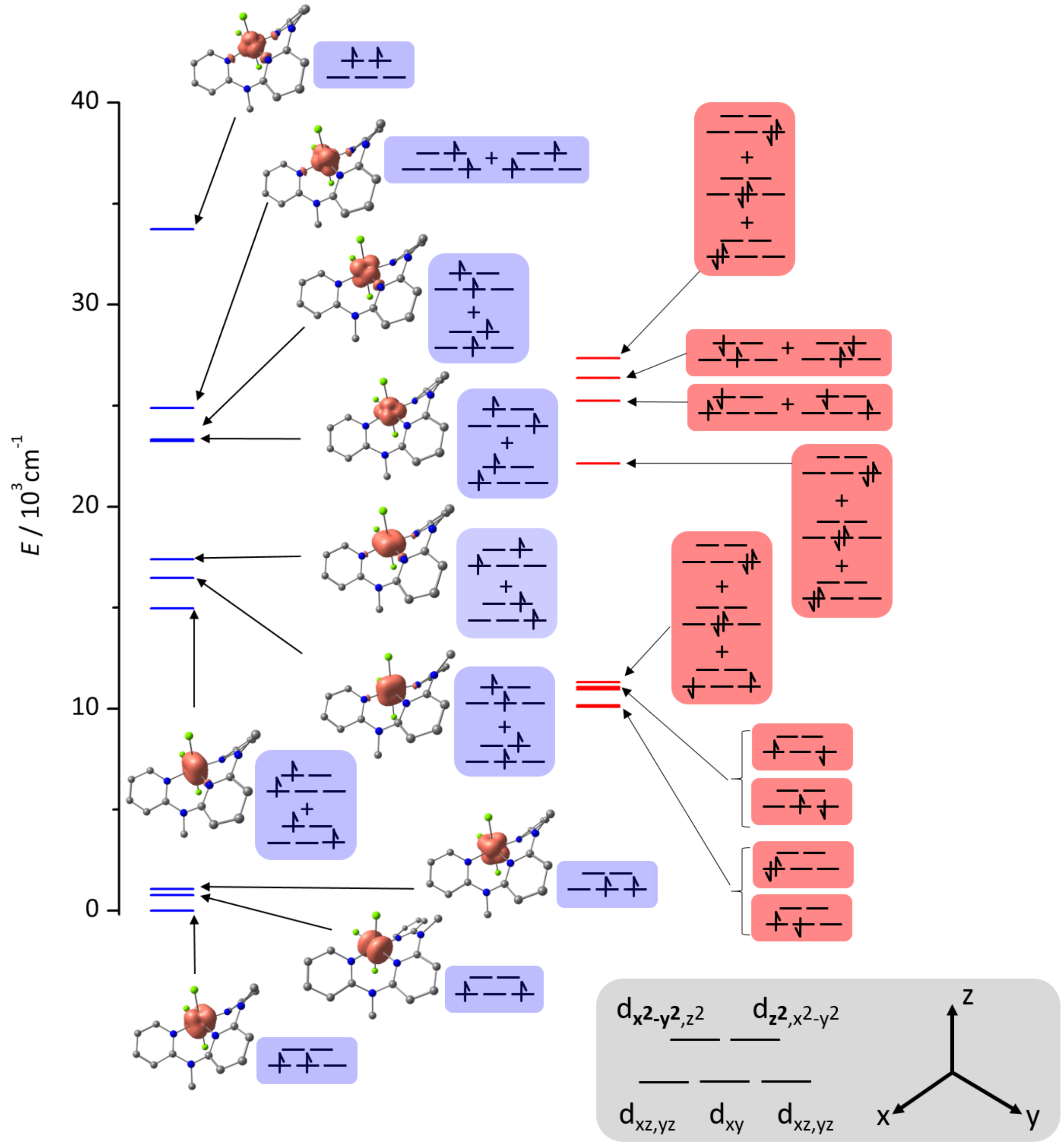

Table S6. Energies of triplet and singlet states/cm^{-1} from the CASSCF(6,12) calculations without and with NEVPT2 (assignments according to the dominant contribution).

state	*E* / cm^{-1} without NEVPT2	*E* / cm^{-1} with NEVPT2	Δ*E* / cm^{-1}
GS ($^3T_1$)	574.7	755.4	180.7
GS ($^3T_1$)	874.1	1076.3	202.2
^1E	12658.8	10085.9	–2572.9
$^1T_2$	12804.9	10160.5	–2644.4
$^1E/^1T_2$	13456.4	10949.1	–2507.3
$^1E/^1T_2$	13580.0	11068.3	–2511.7
$^1E/^1T_2$	13837.7	11309.1	–2528.6
$^3T_2$	13857.6	14967.5	1109.9
$^3T_2$	15252.7	16463.5	1210.8
$^3T_2$	16114.6	17393.1	1278.5
$^1A_1$	26337.3	22141.7	–4195.6
$^3T_1$(P)	25522.4	23253.9	–2268.5
$^3T_1$(P)	25841.4	23318.7	–2522.7
$^3T_1$(P)	27384.5	24890.9	–2493.6
$^1T_2$(G)	27538.6	25238.5	–2300.1
$^1T_2$(G)	28592.3	26376.7	–2215.6
$^1T_2$(G)	29508.1	27339.6	–2168.5

Figure S4. Displacement vectors of Cl–V–Cl deformation vibrations calculated by DFT at 125, 136, 159 cm^{-1} (unscaled).

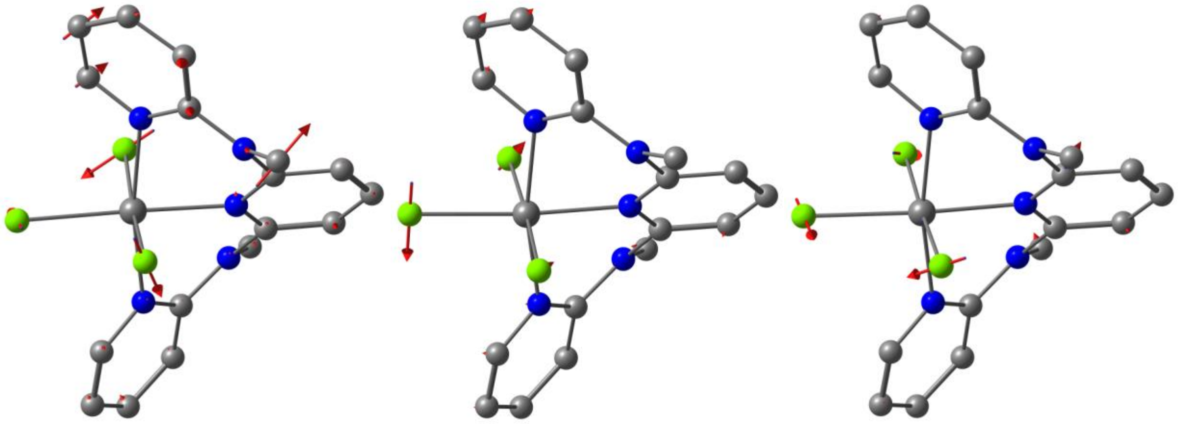

Figure S5. (a) NIR emission bands (peak 1 and 2) of solid $VCl_3(D_0\text{-ddpd})$ under hydrostatic pressure and (b) corresponding peak shift (peak 1 and 2) versus pressure plots.

(a)

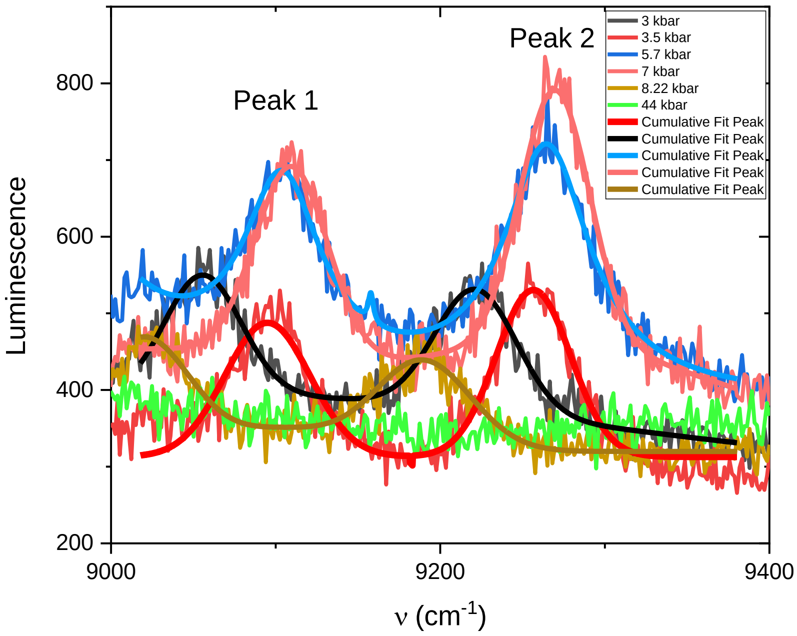

(b)

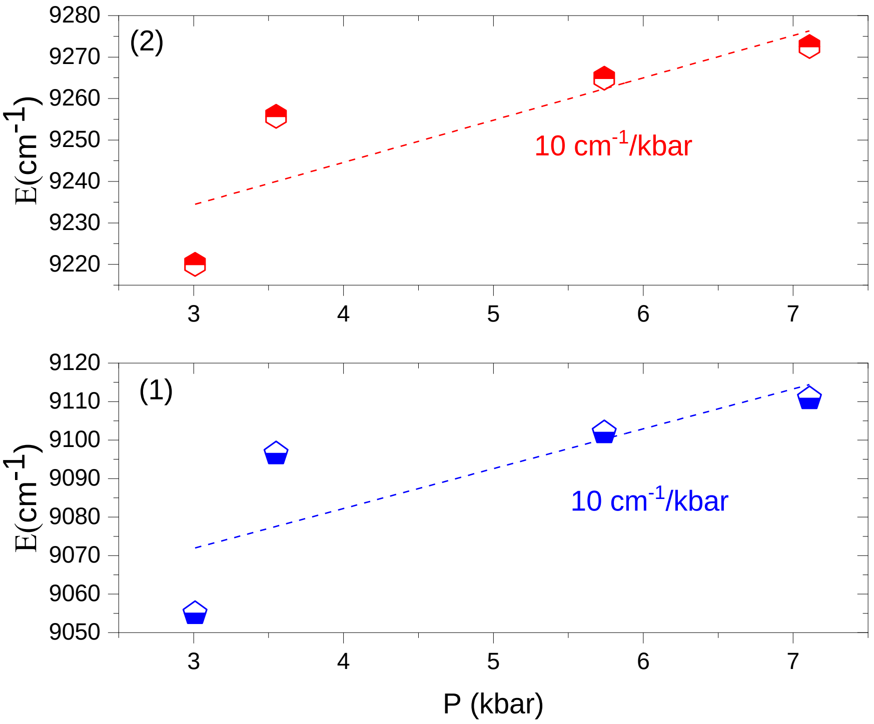

Figure S6. Raman spectra of solid $VCl_3(D_0\text{-ddpd})$ under hydrostatic pressure (λ_{exc} = 785.0 nm).

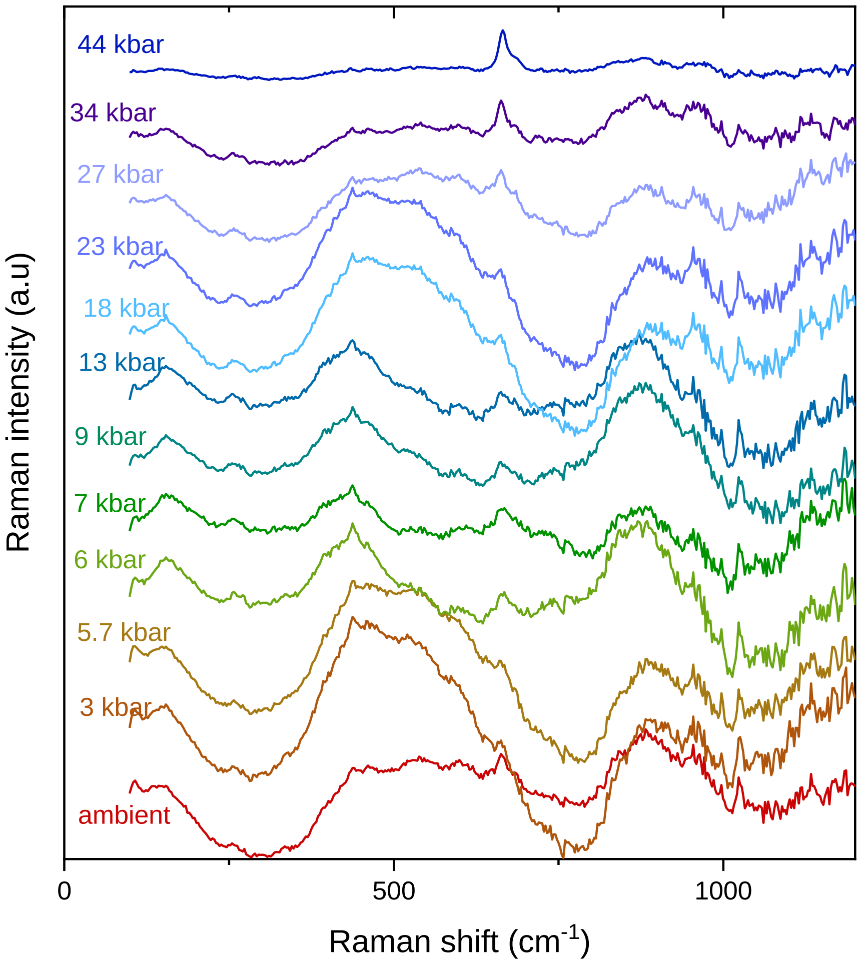

At 44 kbar, the highest pressure achieved for this study, only the vibrational peaks at approximately 665 cm^{-1} are observed, documenting that the electronic Raman signals, dominant at lower pressure, are broadened out to the extreme so that they are no longer clearly identifiable.

Figure S7. Luminescence decay (black scatter) for **VCl_3(ddpd-[D_0])** in the solid state under a dry and deoxygenated argon atmosphere (λ_{exc} = 350 nm, λ_{em} = 1106 nm, excitation path: band pass filter UG11, emission path: long pass filter RG850) with monoexponential fit function (red) and instrument response function (grey).

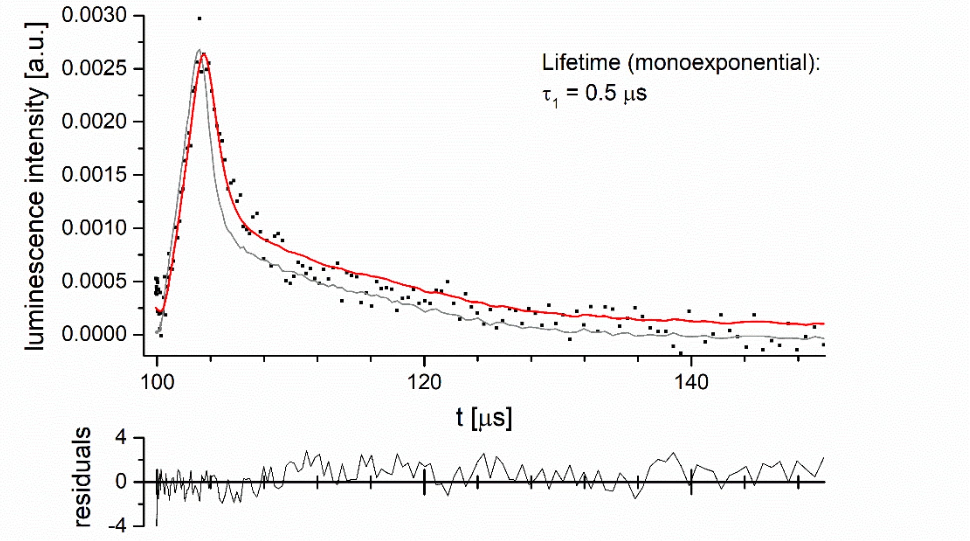

Figure S8. Luminescence decay (black scatter) for **VCl_3(ddpd-[D_{17}])** in the solid state under a dry and deoxygenated argon atmosphere (λ_{exc} = 350 nm, λ_{em} = 1106 nm, excitation path: band pass filter UG11, emission path: long pass filter RG850) with monoexponential fit function (red) and instrument response function (grey).

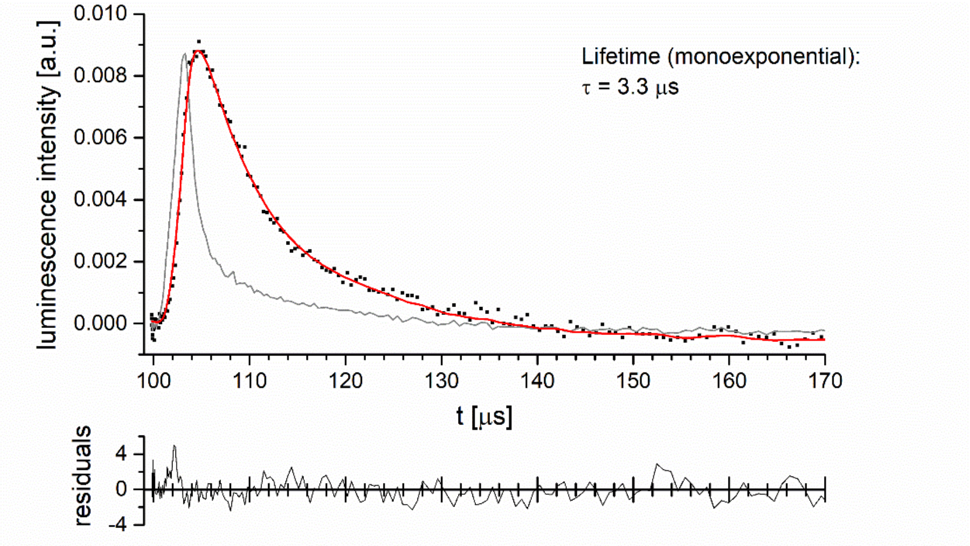

Figure S9. Luminescence decay (black scatter) for **VCl_3(ddpd-[D_{17}])** in the solid state under a dry and deoxygenated argon atmosphere (λ_{exc} = 350 nm, λ_{em} = 1222 nm, excitation path: band pass filter UG11, emission path: long pass filter RG850) with monoexponential fit function (red) and instrument response function (grey).

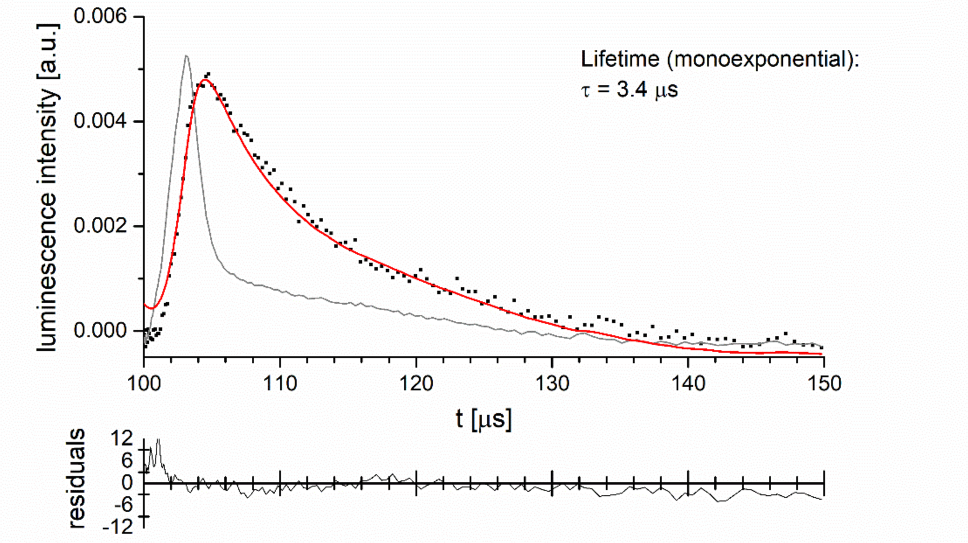

NIR Absorption Spectroscopy / Vibrational Overtone Analysis

Since the isotopologic vanadium(III) complexes were not available in sufficient quantities, the analysis of the position and intensity of the C–H and C–D vibrational overtones for the vanadium complexes was carried out on simpler pyridine-containing model compounds. The vibrational signatures of the protiated and deuterated methyl protons in ddpd were not investigated due to insufficient quantities and because deuteration did not show an effect on the non-radiative deactivation of the metal centered emission of $[Cr(ddpd)_2]^{3+}$.[S3] Previously, the vibrational overtone data of the isotopologues of 6,6'-dimethyl-2,2'-bipyridine (Me_2-bpy and [D_{12}]-Me_2-bpy, Figure S11)[S2] proved to be a successful model system for the analysis of the vibrational overtone structure of ddpd.[S3] For the present analysis, we used the previously obtained aromatic C–(H/D) overtone bands (Gaussians)[S2] of Me_2-bpy and [D_{12}]-Me_2-bpy for the calculations of the spectral overlap integral (SOI) between these oscillators and the vanadium emission.

Figure S10. Comparison of ddpd with the model compounds Me_2-bpy and [D_{12}]-Me_2-bpy used for the analysis of the vibrational C–H and C–D overtones.

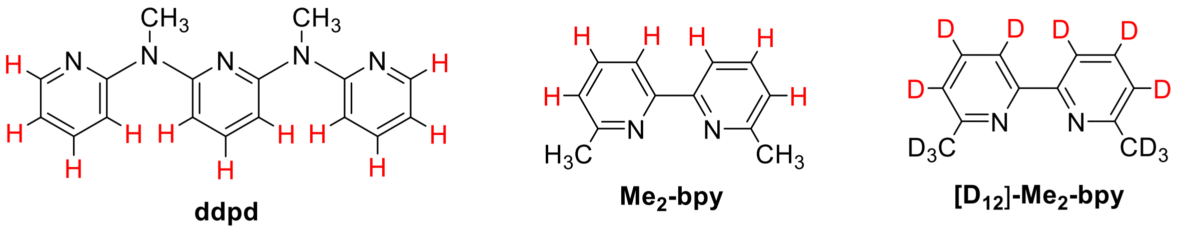

Figure S11. Reconstructed vibrational overtone absorption bands (ν = 2, 3, and 4) for the aromatic C–H oscillators of Me_2-bpy (black) with single Gaussian fits (dashed red).[S3]

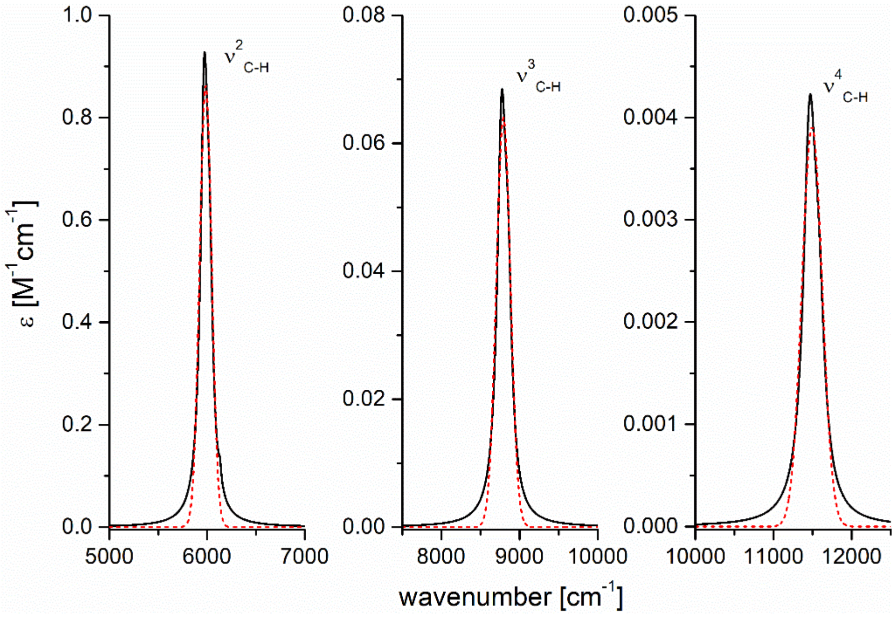

Figure S12. Reconstructed vibrational overtone absorption bands (ν = 3 and 4) for the aromatic CD oscillators of Me_2-bpy (black) with single Gaussian fits (dashed red).[S3]

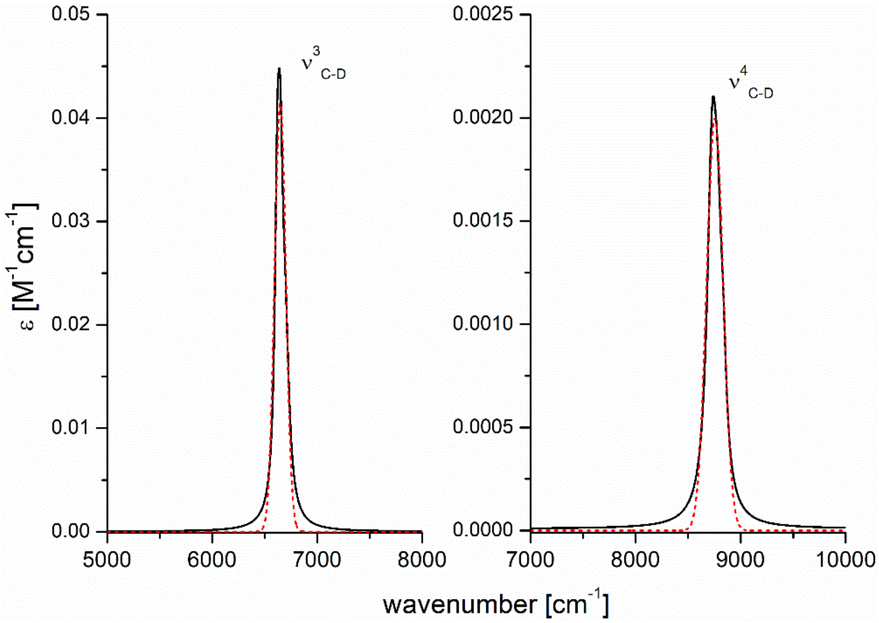

The integrand functions for the SOIs between the vanadium emission and the relevant C–(H/D) overtones were constructed according to the mathematical definition of the SOIs:

$$\mathrm{SOI} = \int I_{\mathrm{norm}}(\tilde{\nu}) \cdot \varepsilon_{\mathrm{vib}}(\tilde{\nu}) \cdot \tilde{\nu}^{-4} d\tilde{\nu}$$

with I_{norm} being the vanadium emission spectrum (normalized to unit area) and ε the molar vibrational extinction coefficient (extracted and extrapolated absorption spectra of the relevant overtones), both expressed in the wavenumber scale $\tilde{\nu}$. The integrand functions were generated with a set of data points with a step size of 1 cm^{-1}. Numerical integration gave the corresponding values for SOI.

Figure S13. Integrand function of the spectral overlap integral (SOI) for the vanadium emission band in VCl_3(ddpd) and the second aromatic C–H (ν = 3) oscillators in Me_2-bpy.

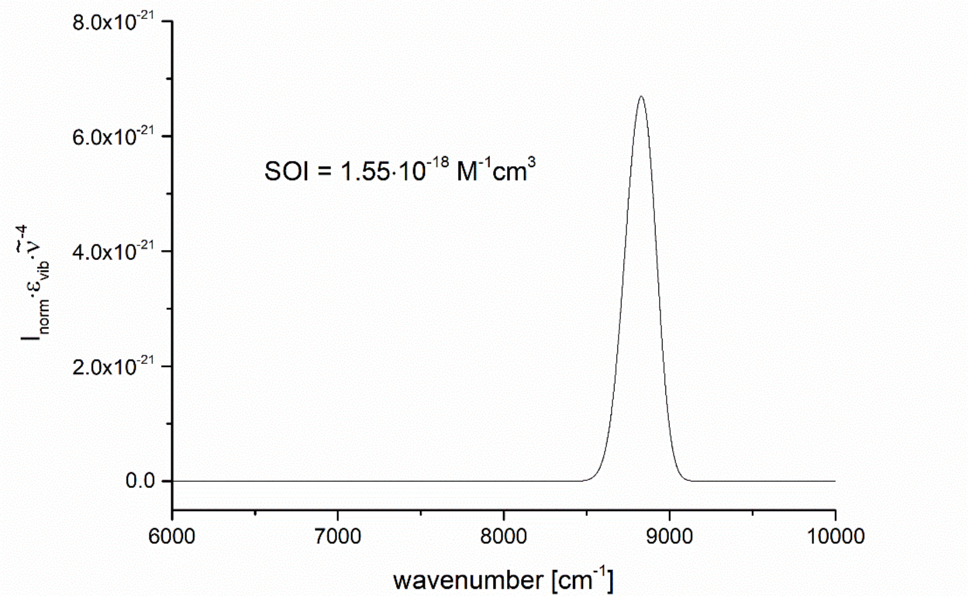

Figure S14. Integrand function of the spectral overlap integral (SOI) for the vanadium emission band in VCl_3(ddpd) and the second (ν = 3) and third (ν = 4) aromatic C–D oscillators in [D_{12}]-Me_2-bpy.

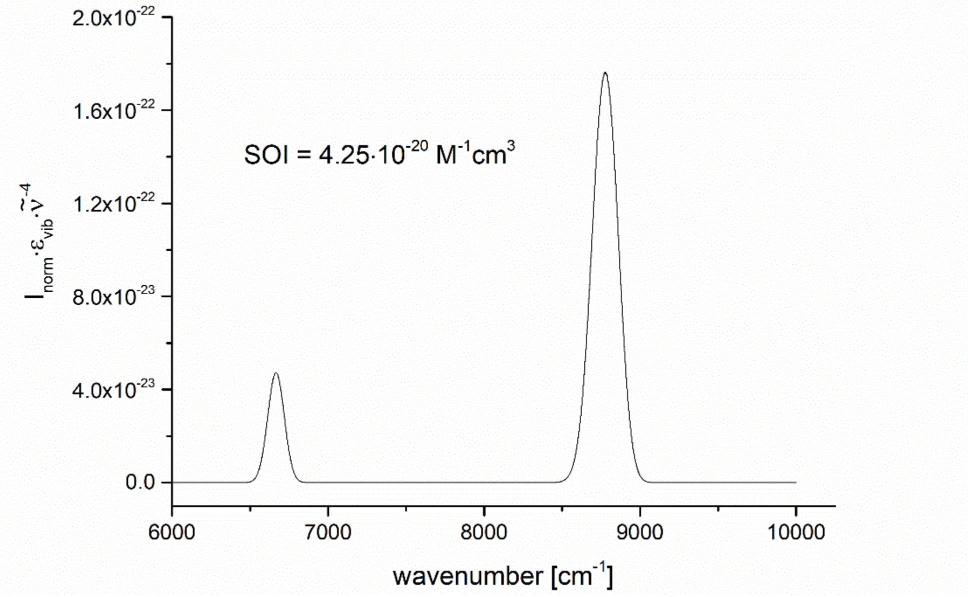

Based on the obtained SOIs, the ratio of the non-radiative deactivation rates of vanadium excited states from VCl_3(ddpd-[D_0]) and VCl_3(ddpd-[D_{17}]) is estimated as:

$$\frac{k_{nr}(\text{C-H})}{k_{nr}(\text{C-D})} = \frac{\text{SOI(C-H)}}{\text{SOI(C-D)}} = \frac{1.55 \cdot 10^{-18}\ \text{M}^{-1}\text{cm}^3}{4.25 \cdot 10^{-20}\ \text{M}^{-1}\text{cm}^3} = 3.63\ \cdot 10^1$$

Figure S15. Normalized spectra for the vanadium ^{1}E emission of VCl_3(ddpd) (red) and the relevant vibrational aromatic C–H overtone absorption band (dashed black).

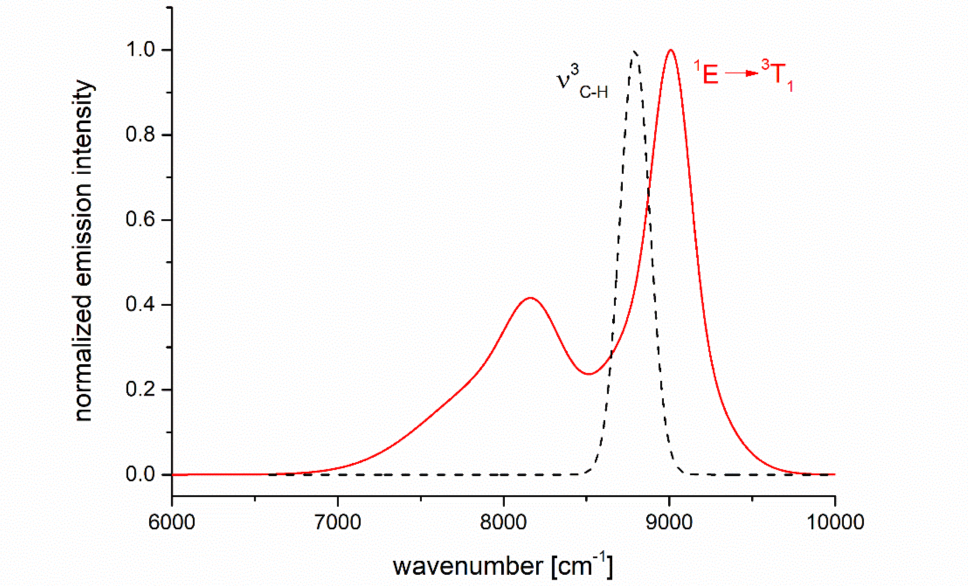

Figure S16. Spectra for the vanadium ^{1}E emission of VCl_3(ddpd) (red) and the relevant vibrational aromatic C–D overtone absorption bands (dashed black). The overtone intensities are shown with their actual intensity ratios.

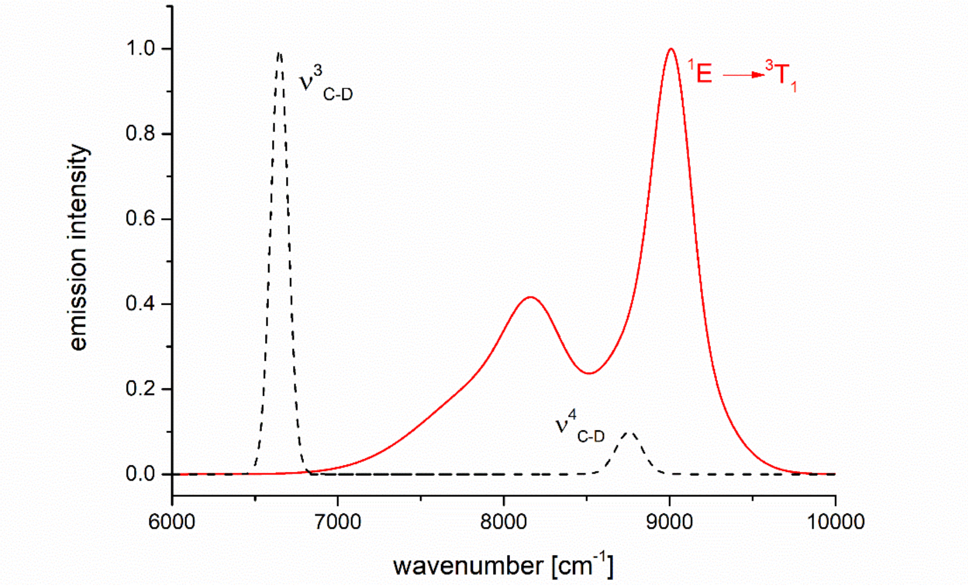

Figure S17. Luminescence spectra of VCl_3(ddpd-[D_0]) as neat powder at 5–290 K (λ_{exc} = 350 nm).

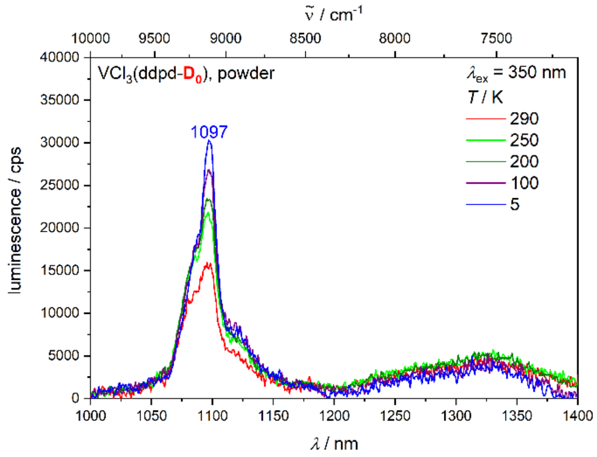

Figure S18. Luminescence spectra of VCl_3(ddpd-[D_0]) as KBr pellet at 5–290 K (λ_{exc} = 350 nm).

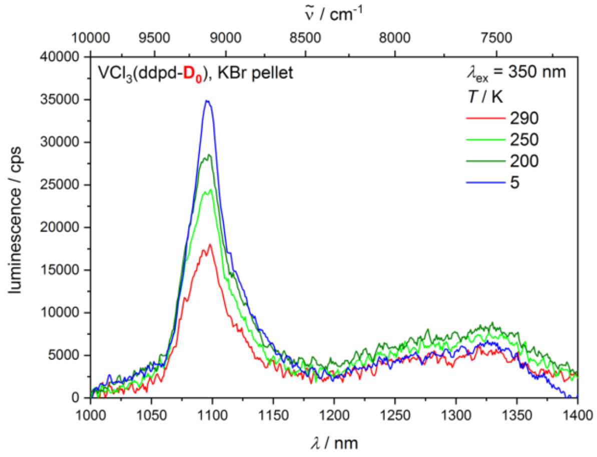

Figure S19. Plot of the integrated luminescence versus temperature for VCl_3(ddpd-[D_0]) and VCl_3(ddpd-[D_{17}]).

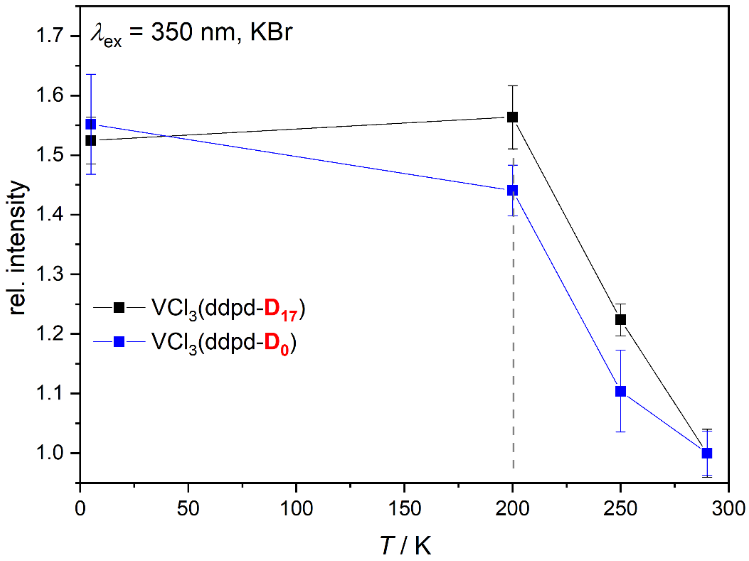

Figure S20. Ground state (black) and step-scan FTIR spectrum (red, λ_{exc} = 355 nm; 0–500 ns) of VCl_3(ddpd-[D_0]) in a KBr pellet at 20 K.

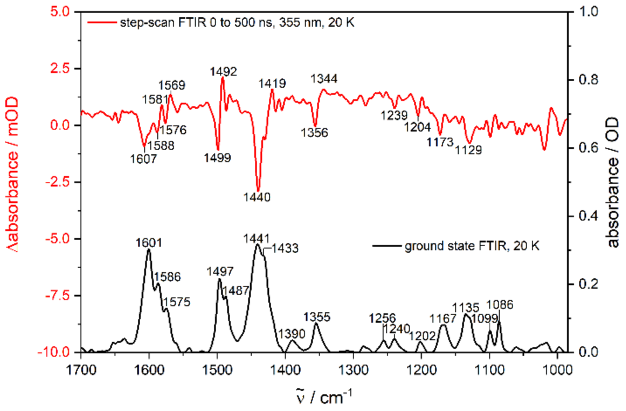

Figure S21. Experimental (black), DFT-calculated ground state (green) and step-scan FTIR spectrum (red, λ_{exc} = 355 nm; 0–500 ns) of VCl_3(ddpd-[D_{17}]) in a KBr pellet at 290 K (red).

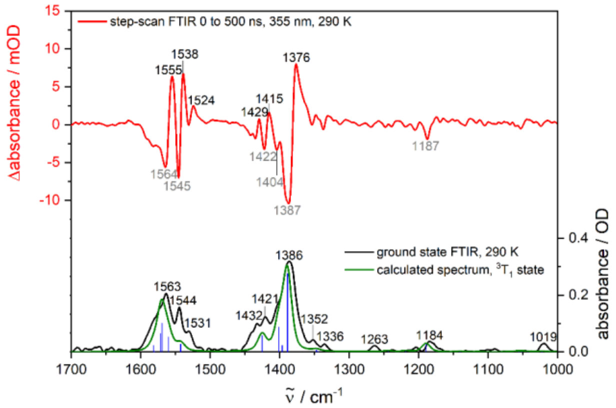

Figure S22. Excited state FTIR spectra of VCl_3(ddpd-[D_{17}]) in a KBr pellet obtained from step-scan FTIR spectra (λ_{exc} = 355 nm; 0–500 ns) (and small contributions of the respective ground state spectrum of 3 %) at 20 K (blue) and 290 K (red).

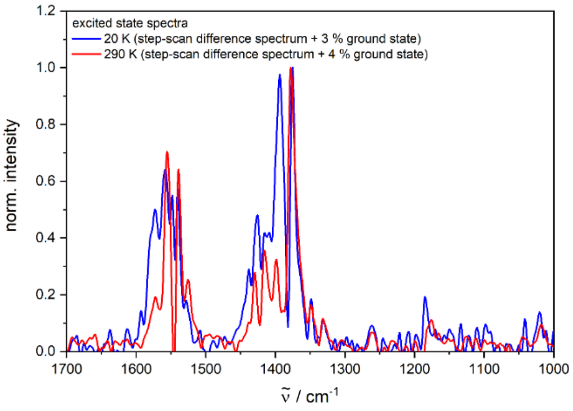

Figure S23. Ground state (black) and step-scan FTIR spectrum (red, λ_{exc} = 355 nm; 0–500 ns) of VCl_3(ddpd-[D_{17}]) in a KBr pellet at 20 K.

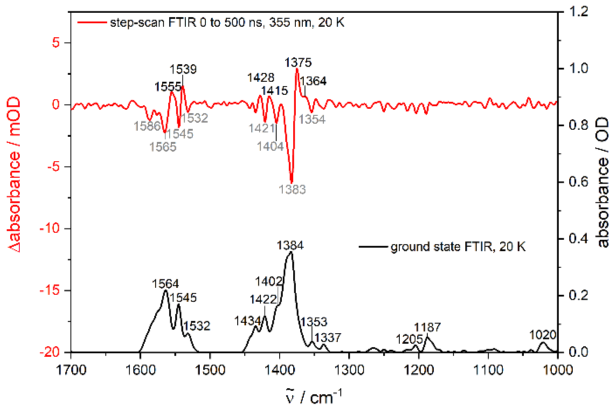

Figure S24. Global monoexponential fit and residuals performed for the most prominent positive and negative peaks in the step-scan spectrum of VCl_3(ddpd-[D_0]) in a KBr pellet at 290 K.

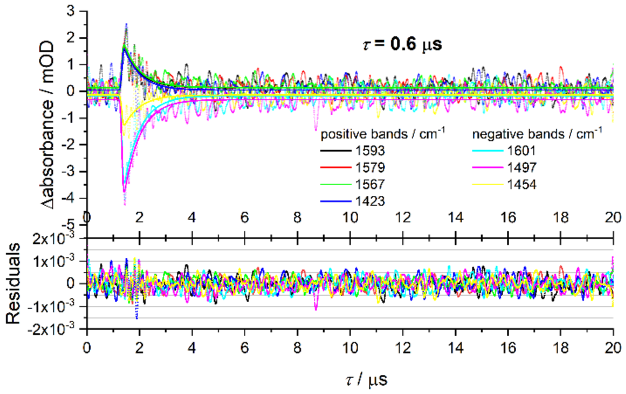

Figure S25. Global monoexponential fit and residuals performed for the most prominent positive and negative peaks in the step-scan spectrum of VCl_3(ddpd-[D_0]) in a KBr pellet at 20 K.

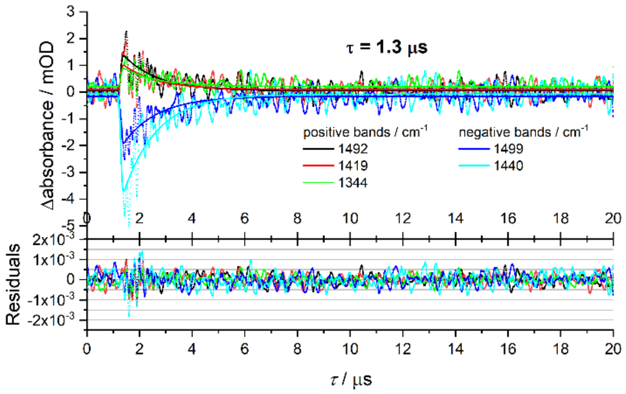

Figure S26. Global monoexponential fit and residuals performed for the most prominent positive and negative peaks in the step-scan spectrum of VCl_3(ddpd-[D_{17}]) in a KBr pellet at 290 K.

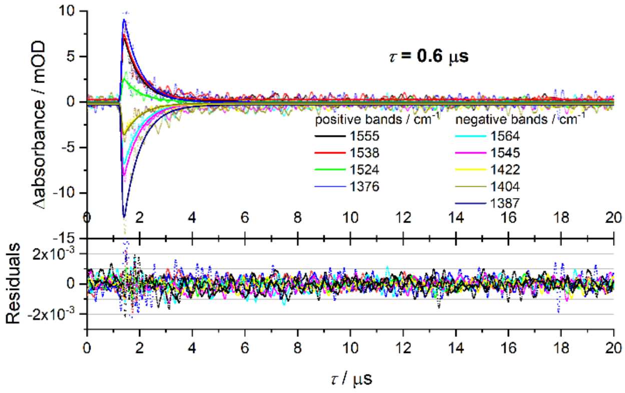

Figure S27. Global monoexponential fit and residuals performed for the most prominent positive and negative peaks in the step-scan spectrum of VCl_3(ddpd-[D_{17}]) in a KBr pellet at 20 K.

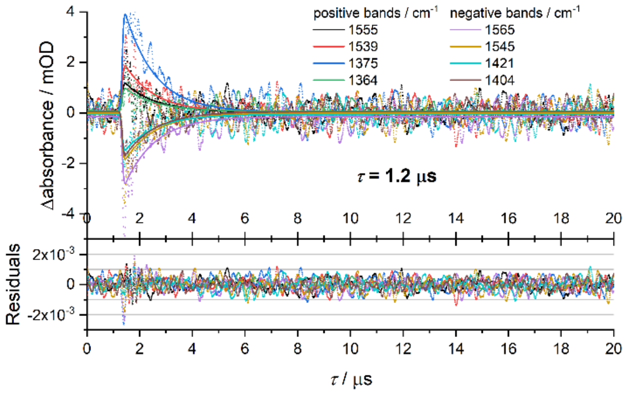

Figure S28. Changes of the UV/Vis absorption spectra of VCl_3(ddpd-[D_0]) in CH_3CN under irradiation with a Xe lamp at 350±5 nm.

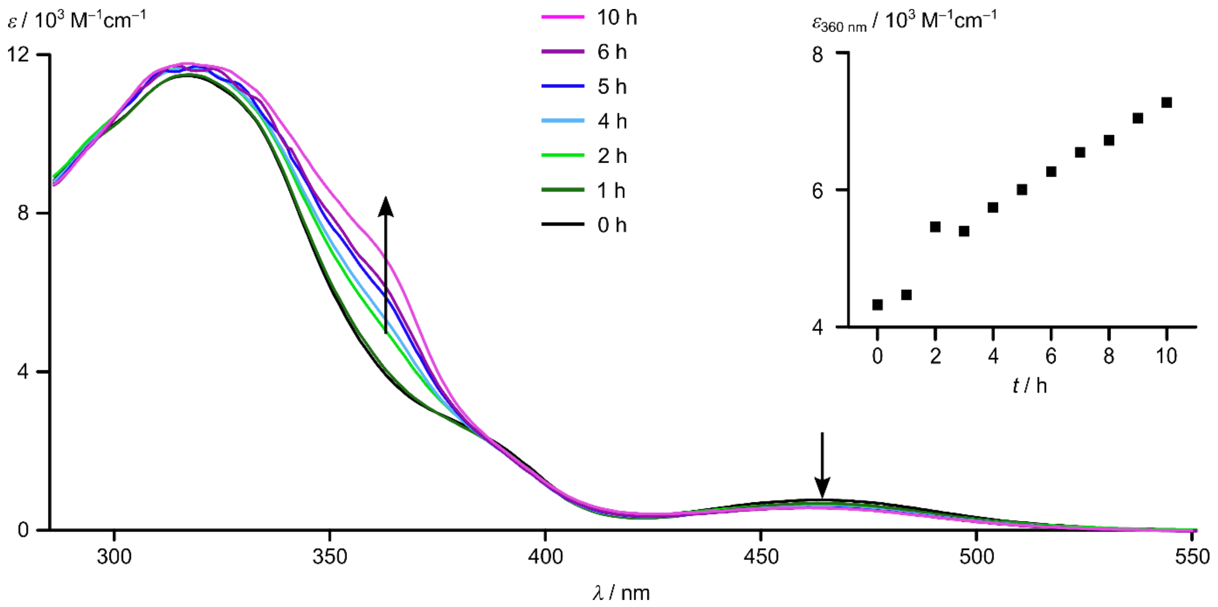

Figure S29. Changes of the emission spectra of VCl_3(ddpd-[D_0]) in CH_3CN under irradiation with a Xe lamp at 350±5 nm.

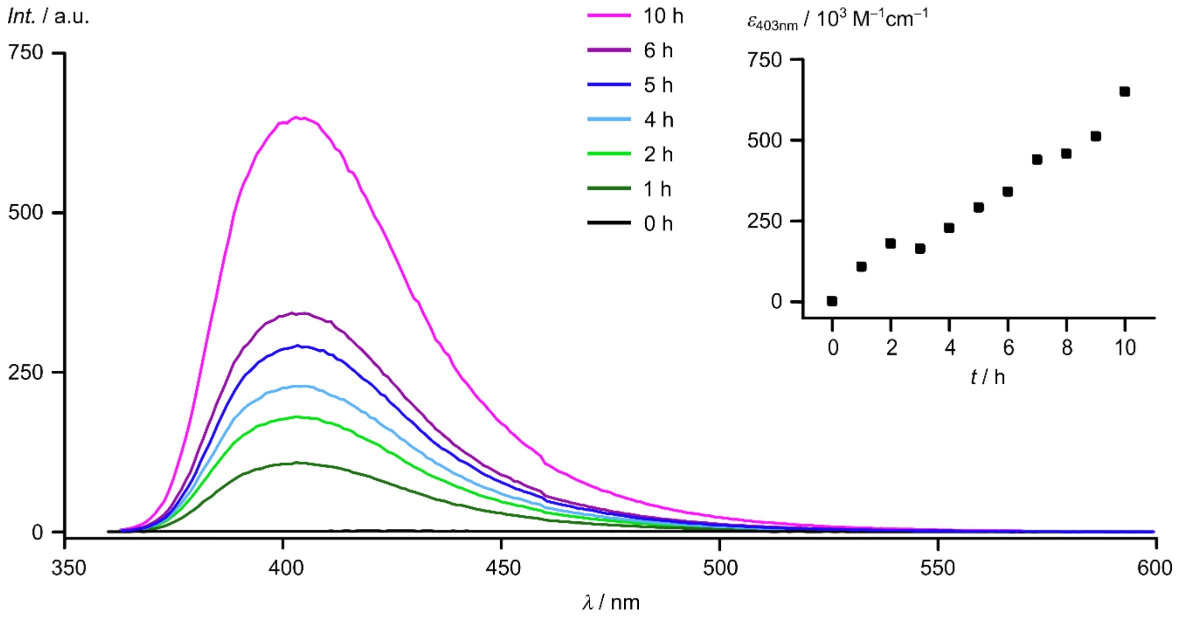

Figure S30. Changes of the UV/Vis absorption spectra of VCl_3(ddpd-[D_0]) in CH_3CN under irradiation with a Xe lamp at 400±5 nm.

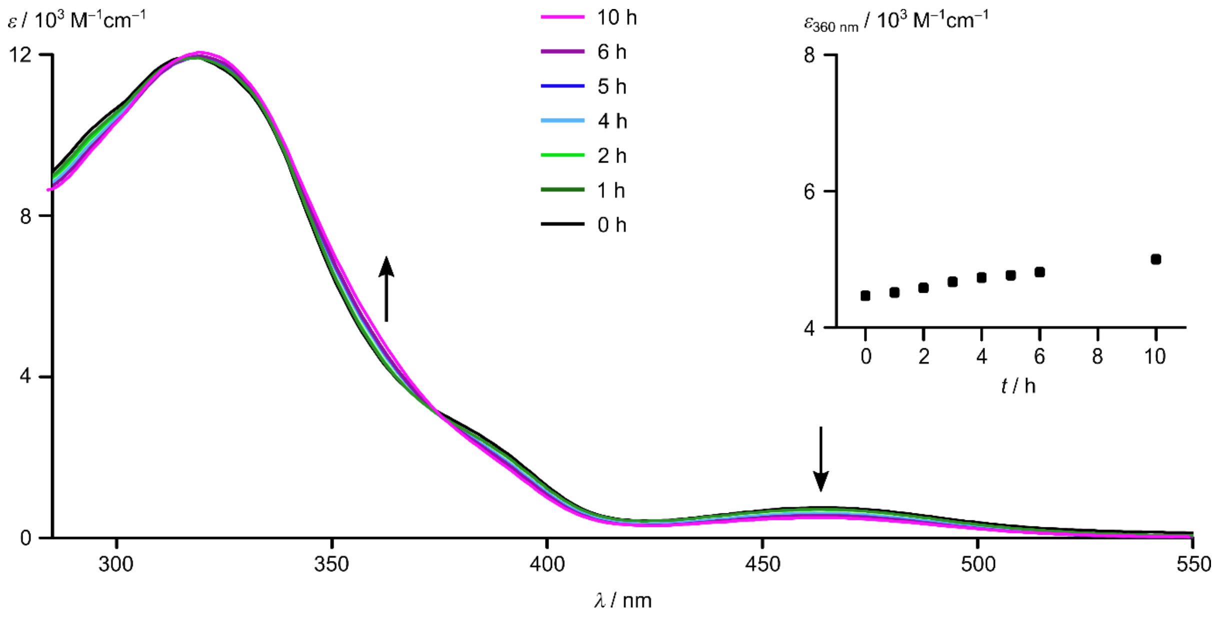

Figure S31. Changes of the emission spectra of VCl_3(ddpd-[D_0]) in CH_3CN under irradiation with a Xe lamp at 400±5 nm.

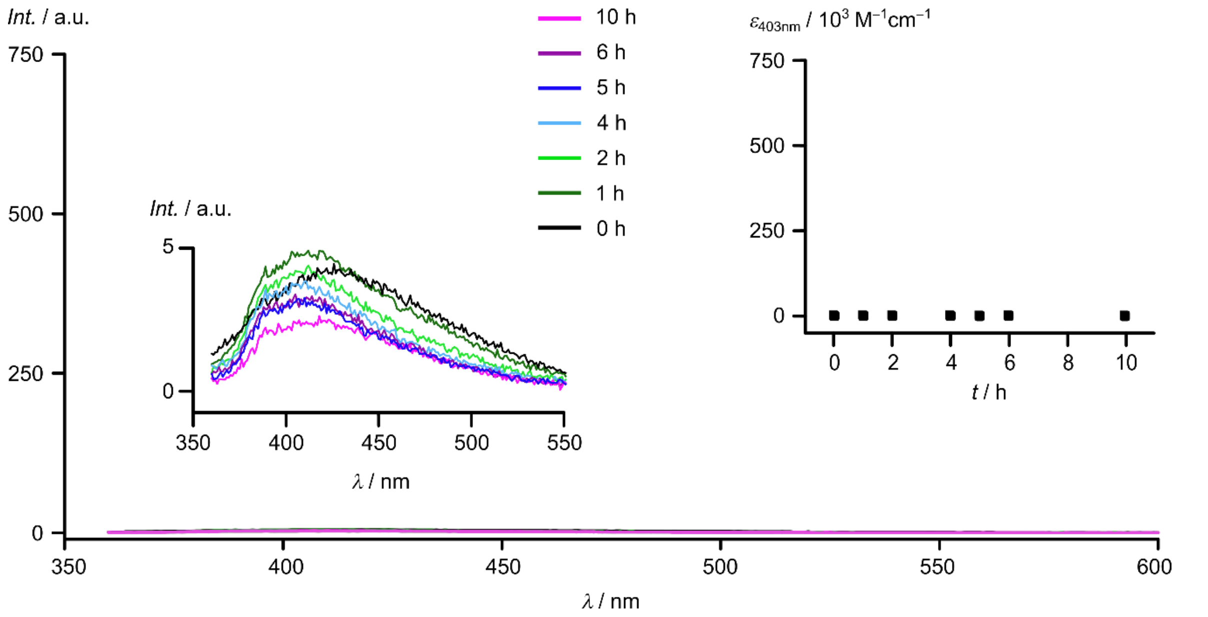

Figure S32. Spectral irradiance of the employed light source Asahi Spectra Max-303 Xenon Light Source (300 W).

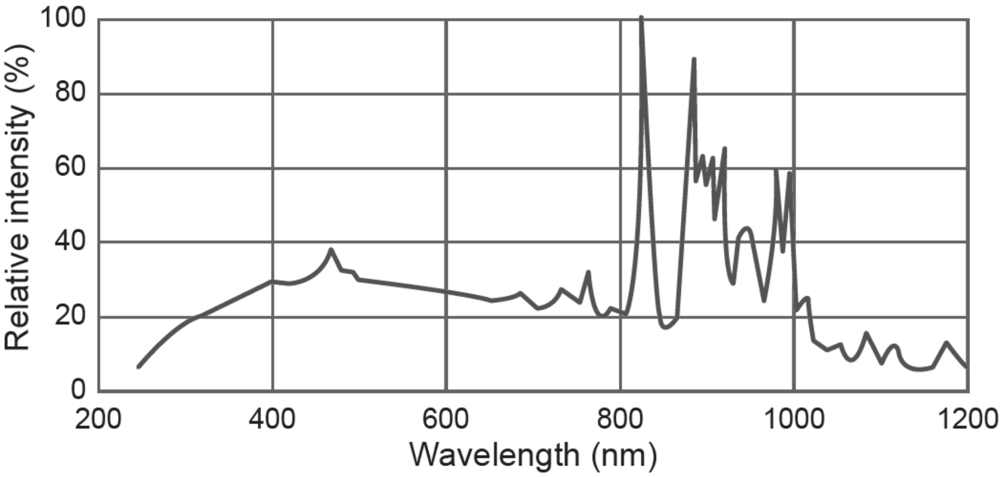

Figure S33 Cyclic voltammograms of VCl_3(ddpd) at 298 K in [$^{n}Bu_4N$][PF_6]/CH_3CN. Potentials given vs. ferrocene/ferrocenium.

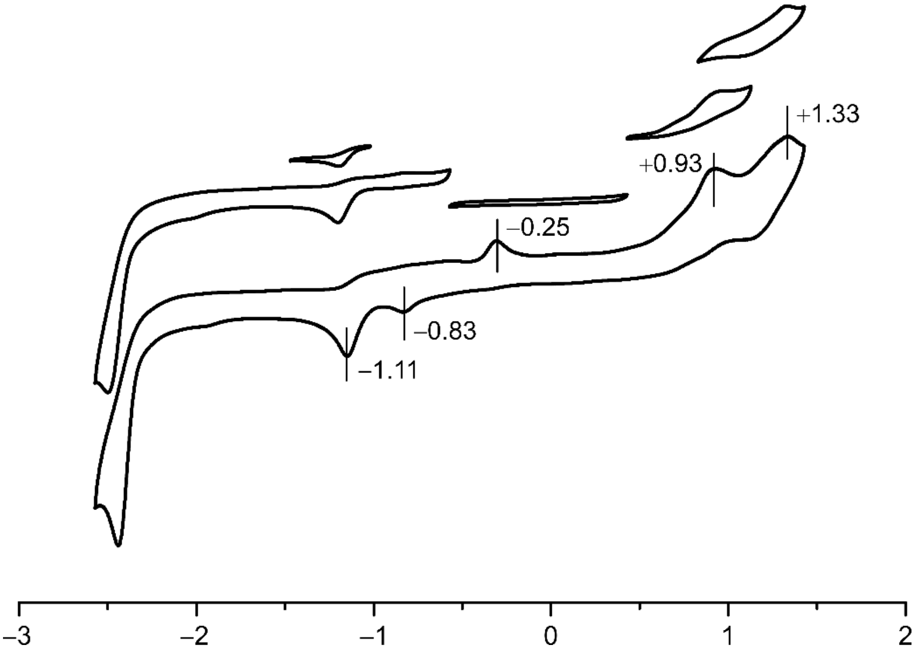

References

S1 C. Förster, M. Dorn, T. Reuter, S. Otto, G. Davarci, T. Reich, L. Carrella, E. Rentschler and K. Heinze, *Inorganics* 2018, **6**, 86.

S2 C. Doffek, N. Alzakhem, C. Bischof, J. Wahsner, T. Güden-Silber, J. Lügger, C. Platas-Iglesias and M. Seitz, *J. Am. Chem. Soc.* **2012**, *134*, 16413−16423.

S3 C. Wang, S. Otto, M. Dorn, E. Kreidt, J. Lebon, L. Sršan, P. Di Martino-Fumo, M. Gerhards, U. Resch-Genger, M. Seitz and K. Heinze, *Angew. Chem.* **2018**, *130*, 1125–1130.
